# Oriented Cortical‐Bone‐Like Silk Protein Lamellae Effectively Repair Large Segmental Bone Defects in Pigs

**DOI:** 10.1002/adma.202414543

**Published:** 2025-01-28

**Authors:** Yajun Shuai, Tao Yang, Meidan Zheng, Li Zheng, Jie Wang, Chuanbin Mao, Mingying Yang

**Affiliations:** ^1^ Institute of Applied Bioresource Research College of Animal Sciences Zhejiang University Hangzhou 310058 China; ^2^ Key Laboratory of Silkworm and Bee Resource Utilization and Innovation of Zhejiang Province Zhejiang University Hangzhou 310058 China; ^3^ Department of Biomedical Engineering The Chinese University of Hong Kong Sha Tin Hong Kong SAR China; ^4^ Guangxi Engineering Center in Biomedical Materials for Tissue and Organ Regeneration & Guangxi Collaborative Innovation Center for Biomedicine Life Sciences Institute Guangxi Medical University Nanning 530021 China

**Keywords:** bio‐manufacturing, bone tissue regeneration, cortical bone organoids, freeze‐casting, silk fibroin

## Abstract

Assembling natural proteins into large, strong, bone‐mimetic scaffolds for repairing bone defects in large‐animal load‐bearing sites remain elusive. Here this challenge is tackled by assembling pure silk fibroin (SF) into 3D scaffolds with cortical‐bone‐like lamellae, superior strength, and biodegradability through freeze‐casting. The unique lamellae promote the attachment, migration, and proliferation of tissue‐regenerative cells (e.g., mesenchymal stem cells [MSCs] and human umbilical vein endothelial cells) around them, and are capable of developing in vitro into cortical‐bone organoids with a high number of MSC‐derived osteoblasts. High‐SF‐content lamellar scaffolds, regardless of MSC inoculation, regenerated more bone than non‐lamellar or low‐SF‐content lamellar scaffolds. They accelerated neovascularization by transforming macrophages from M1 to M2 phenotype, promoting bone regeneration to repair large segmental bone defects (LSBD) in minipigs within three months, even without growth factor supplements. The bone regeneration can be further enhanced by controlling the orientation of the lamella to be parallel to the long axis of bone during implantation. This work demonstrates the power of oriented lamellar bone‐like protein scaffolds in repairing LSBD in large animal models.

## Graphical abstract

Silk proteins can be fabricated into large scaffolds with cortical‐bone‐like lamellar structures through freeze‐casting. The resultant freeze‐cast scaffolds are made of parallel silk protein lamellae and can be implanted into pig models to achieve effective bone regeneration without side effects.

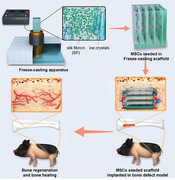

## 1 Introduction

Bone defects result from various causes (e.g., tumor removal, infections, metabolic diseases, trauma, or congenital malformations). Those that exceed a critical size cannot naturally heal and are termed a large segmental bone defect (LSBD).^[^ ^1^ ^]^ The effective repair of LSBD and restoration of the physiological functions of bone tissue pose major challenges in orthopedic surgery. Current clinical treatments for LSBD include autogenous or heterologous bone grafts,^[^ ^2^ ^]^ distraction osteogenesis,^[^ ^3^ ^]^ gene therapy,^[^ ^4^ ^]^ and periosteum‐induced osteogenesis.^[^ ^5^ ^]^ However, these approaches have inherent limitations, such as limited availability of sources, requirement for multiple surgeries, increased surgical trauma and pain, and risk of severe immune rejection, and have never been effective in repairing LSBD.^[^ ^6^ ^]^

Tissue engineering therapy, which utilizes artificial 3D biomaterials with extracellular matrix (ECM) properties, has emerged as a promising treatment for bone defects.^[^ ^1^, ^7^ ^]^ Biocompatible artificial scaffolds can be prepared using bio‐mild polymer materials through freeze drying, gas foaming,^[^ ^8^ ^]^ and sintering process.^[^ ^1b^ ^]^ However, the pore interconnectivity of these prepared 3D scaffolds is generally irregular and poor, significantly different from the microstructure of natural bone tissue. As a result, these scaffolds exhibit insufficient cell recruitment, vascularization, and osteoinduction after in vivo implantation. Therefore, to enhance functionality, growth factors such as RGD,^[^ ^9^ ^]^ vascular endothelial growth factor (VEGF),^[^ ^10^ ^]^ bone morphogenetic protein 2 (BMP2),^[^ ^11^ ^]^ calcium phosphate,^[^ ^1b^ ^]^ or bioactive elements^[^ ^12^ ^]^ need to be added to ensure the ability to repair LSBD. However, this introduces new challenges, including high cost, inaccurate in vivo release, toxicity, and rapid metabolism of exogenous growth factors in vivo.

Human bone can be divided into cortical bone and cancellous bone, with the former accounting for 80% of all bone mass. The cortical bone is composed of micro‐level bone units known as the Haversian system. These units contain 4–20 parallel layers of bone lamella,^[^ ^13^ ^]^ which provide ample living space for cells, facilitate vascular permeability, and exhibit anisotropic mechanical properties. Recently, significant advancements have been made in utilizing 3D printing technology to create bone‐like structures and intricate geometries to promote bone tissue regeneration,^[^ ^14^ ^]^ holding the prospect of repairing LSBD.^[^ ^9^, ^15^ ^]^ However, 3D printing technology still faces challenges, such as developing a biosafe printing ink, enhancing printing resolution (which is often below 50 µm), and ensuring the stability of printed structures. Consequently, there is a strong motivation to prepare 3D scaffolds with highly organized micro‐lamellar structures and stable frameworks to enhance cell recruitment and bone repair without the addition of growth factors.

Since natural proteins are biocompatible, scaffolds made of natural proteins such as silk fibroin (SF) or collagen are attractive candidates for bone regeneration.^[^ ^7^, ^16^ ^]^ There is a high demand for assembling natural proteins into a large scaffold with superior mechanical properties and bone‐like microstructures that can be used in repairing large defects (e.g., LSBD) at the load‐bearing sites in large animals (e.g., pigs or sheep) and even primates. Although multiple approaches have been tried such as 3D printing and freeze‐drying,^[^ ^17^ ^]^ assembling natural proteins into the desired scaffolds for repairing bone defects in large animal models is still challenging.

Meanwhile, freeze‐casting is a 3D material processing technology that uses ice crystals as a template to process polymers,^[^ ^18^ ^]^ and carbon‐based materials^[^ ^19^ ^]^ into an anisotropic structure with orderly geometries, such as multi‐layer lamella, grids, or honeycombs. This technology has important applications in battery electrodes, flame retardant materials, environmental applications, and filtering materials.^[^ ^20^ ^]^ Although there have been some studies exploring the use of oriented silk protein scaffolds for biomedical use,^[^ ^21^ ^]^ there remains a significant gap regarding their application in biomimetic cortical‐bone organoids and their use in studies of large animal models for LSBD regeneration. Until now, the preparation and application of pure protein‐based 3D scaffolds with highly biomimetic bone lamella microstructures by freeze‐casting have not been thoroughly investigated for bone tissue engineering in large animal models.

**Figure 1:**
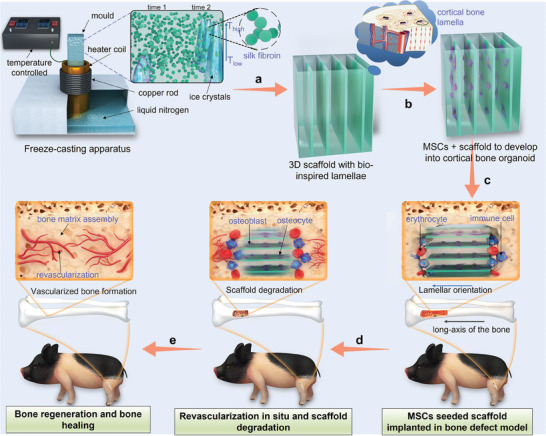
Construction of SF lamellar scaffolds for in vitro 3D cell culture and repair of LSBD in vivo. a) SF scaffolds with bone‐like lamellar structures are prepared by a freeze‐casting instrument, which includes a liquid nitrogen box, a heating coil, a copper rod, and a temperature control system. b) After MSCs are seeded within the SF lamellar scaffold, the MSCs adhere, migrate, and proliferate on the surface of the lamellae. c) MSC‐seeded scaffolds are implanted into an LSBD model in mini‐pig models. d) At the early‐stage post‐implantation, the SF lamellar scaffold recruits inflammatory cells in vivo and promotes vascular regeneration. e) At the later stage post‐implantation, continued degradation of the scaffold and osteogenesis‐angiogenesis coupling promote the maturation of the bone matrix and the regeneration of new bone.

To achieve this goal, we propose to use SF as a material for fabricating freeze‐cast scaffolds with cortical bone‐like lamellae to repair LSBD for three reasons. First, SF has been shown to possess exceptional biocompatibility owing to its amino acid composition, its being a structural protein derived from silkworms, and its high purity. Moreover, SF has been approved for clinical use by the U.S. Food and Drug Administration (FDA) and China's National Medical Products Administration (NMPA), etc.^[^ ^22^ ^]^ Second, SF‐based scaffolds can achieve a controlled degradation rate in vivo by regulating secondary structures, thus facilitating tissue generation.^[^ ^22^ ^]^ Finally, scaffolds originating from SF typically possess outstanding mechanical characteristics.^[^ ^23^ ^]^ Their high resistance to deformation makes SF a preferred biomaterial for load‐bearing composites, enhancing their suitability for bone tissue engineering. We extracted SF from silkworm cocoons using our previously established protocols^[^ ^24^ ^]^ and added it into a self‐made freeze‐casting apparatus. At a constant rate of cooling, ice crystals grew in parallel with proteins repelled into the areas between the ice crystals (**Figure** **1**a). After the sublimation of the parallel ice crystals, the ice crystal area became the pores and the protein area turned into biomaterials, producing SF‐based scaffolds with a unique lamellar structure (Figure 1a). By exploring the casting process, we have identified several parallel lamella structures that are homogeneous, interconnected, suitable for cell infiltration, conducive to long‐term cell survival, and capable of developing into cortical bone organoids (Figure 1b). Due to the green, biodegradable, bioabsorbable properties of SF,^[^ ^24^, ^25^ ^]^ we expected that this bioinspired scaffold, which has a structure highly similar to cortical bone, would be capable of repairing bone defects in an LSBD model of Bama mini pigs without the need of a growth factor in the scaffold (Figure 1c–e).

## 2 Results

### 2.1 Characterization of SF Lamellar Scaffolds

**Figure 2:**
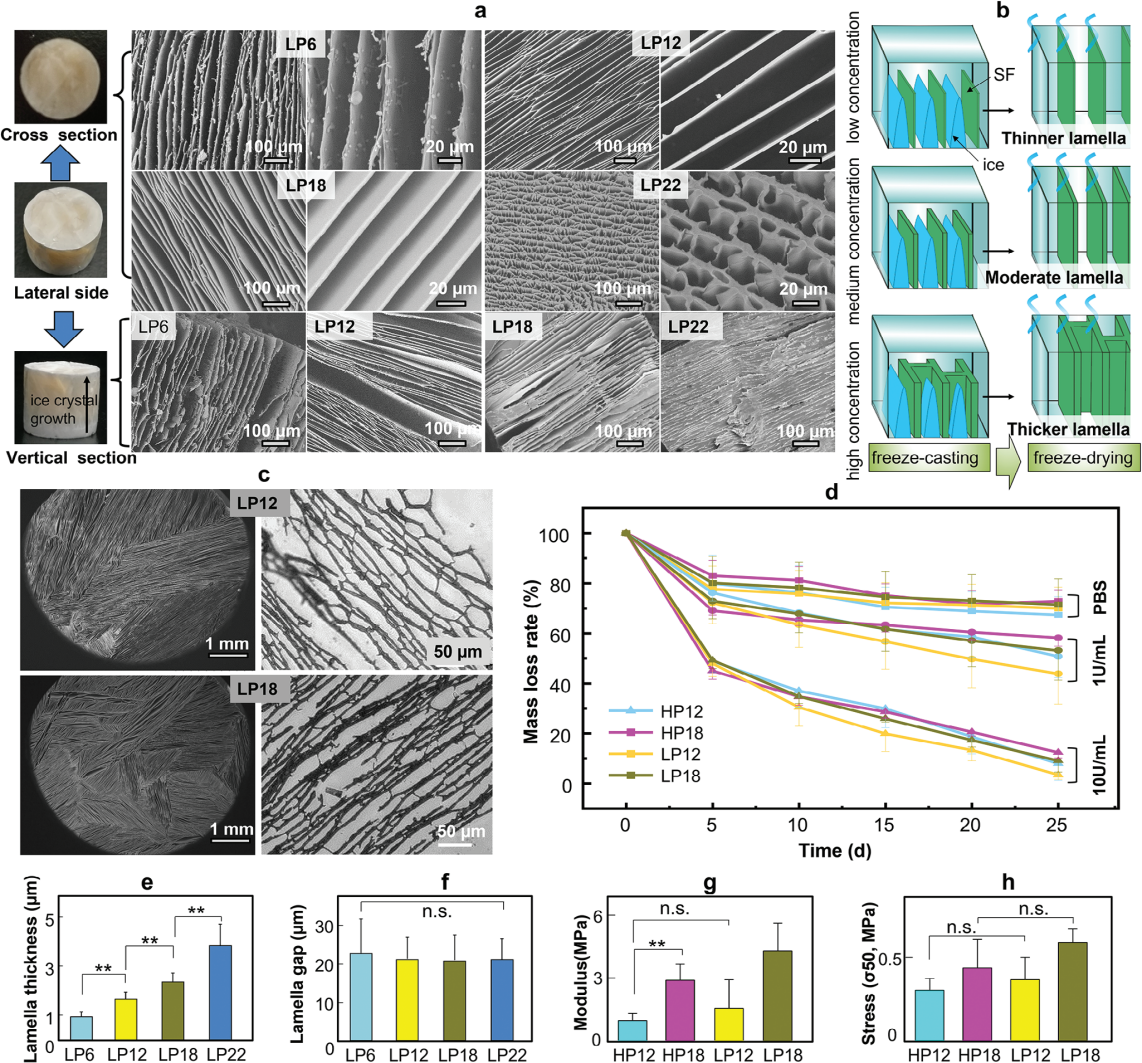
Microarchitecture, mechanical and degradation properties of LP scaffolds. a) SEM view of the cross‐section and vertical sections of LP scaffolds observed. b) The schematic depicts the structure of the thinner lamellae, medium lamellae, and bridging thicker lamellae formed during the freeze‐casting of an SF solution from low to high concentrations. c) SEM of LP scaffolds at low magnification (left panel) and ultra‐thin sections (right panel) of LP scaffolds. d) Degradation profiles of LP scaffolds under oscillatory conditions in phosphate‐buffered saline (PBS) or protease with varying enzyme activity (10 and 1 U mL^−1^). e,f) Quantification of the lamella thickness (e) and the lamella spacing (f) of the LP scaffolds, showing that the lamella thickness of LP scaffolds increases significantly but the lamella spacing of LP scaffolds is nearly unchanged, with the increase in the SF concentrations. Error bars represent the standard deviation (SD) for 45–55 randomly selected regions. g) The compressive modulus of LP scaffolds (*n* = 4). h) σ50 refers to the stress of scaffolds at 50% compression strain (*n* = 4). HP scaffolds: honeycomb porous (HP) scaffolds prepared by only freeze‐drying an SF solution with the concentration of 6% (w/v), 12% (w/v), 18% (w/v), or 22% (w/v). The resultant scaffolds are termed HP6, HP12, HP18, and HP22, respectively. LP scaffolds: lamellar porous (LP) scaffolds prepared by freeze‐casting of an SF solution with the concentration of 6% (w/v), 12% (w/v), 18% (w/v), or 22% (w/v). The resultant scaffolds are termed LP6, LP12, LP18, and LP22, respectively. Bar graphs show mean ± SD. ^**^ *p* < 0.01 indicates significant differences. n.s. denotes no significant difference.

The freeze‐drying method is a commonly used technique for preparing honeycomb‐like porous 3D biomaterials. Here, we first prepared honeycomb‐like porous (HP) scaffolds by this method as the control group (Figure S1, Supporting Information). To replicate the optimal bone lamellar microstructure of natural cortical bone in a laboratory setting, SF scaffolds with a unidirectional lamellar porous (LP) structure were successfully created using another different method, the freeze‐casting (Figures 1a and **2**a). As demonstrated by scanning electron microscope (SEM) imaging of ultrathin sections, the surface and interior of all SF freeze‐cast scaffolds have a homogeneous lamellar structure (Figure 2a,c). When the SF concentration is between 12% and 18%, the microstructure exhibits a highly ordered lamellar structure aligned with the growth direction of ice crystals. Importantly, as the SF concentration increased to 22%, dendritic branch structures appeared between the lamellae, connecting them tightly. The appearance of the dendritic structure was mainly due to the restricted space available for the directional growth of ice crystals following the increase in SF concentration (Figure 2b). Besides, we also observed the longitudinal sections of LP scaffolds and found that the long‐axis (z‐axis) direction of lamellar structures in all LP scaffolds was parallel to each other (Figure 2a), which was highly similar to the osteon microstructure of compact bones. The average lamellar thickness of different LP scaffolds (named LPXX with XX representing the initial SF concentrations), including LP6 (6 w/v%), LP12 (12 w/v%), LP18 (18 w/v%), and LP22 (22 w/v%), increased from 0.9  to 1.7 µm, 2.4 , and 3.8 µm, respectively (Figure 2e; Figure S2, Supporting Information). However, the change in lamellar spacing was not significant with the increase in the SF concentration, due to the thickening of lamellae and the formation of dendritic branch structures (Figure 2b,f).

The mechanical properties of bone‐repairing materials are crucial in maintaining the normal function of osteocytes and promoting effective bone regeneration.^[^ ^26^ ^]^ Our results show that the elastic modulus and compression stress at 50% strain of all LP scaffolds are higher than those of the HP scaffolds at the same SF concentrations (Figure 2g,h). These results indicate that SF scaffolds with homogenous lamellae and the consequent high mechanical strength can be effectively produced by the freeze‐casting technology. Although the specific mechanical properties of the scaffolds intended for the repair of weight‐bearing bone defects, ranging from implantation to complete healing, remain to be fully characterized.^[^ ^27^ ^]^ The LP scaffolds exhibit mechanical properties that fall within an acceptable range relative to those of host bone, thus supporting their potential for bone regeneration.^[^ ^28^ ^]^ Additionally, we conducted in vitro degradation experiments to quantify the degradation rate of scaffolds under controlled conditions. Figure 2d shows that all SF scaffolds degrade over time at a rotational speed of 180 rpm and a temperature of 37 °C. Notably, the degradation rate of the LP scaffolds was faster than that of HP at the same concentration, providing valuable insights into the longevity and performance of these scaffolds in a physiological environment.

### 2.2 Long‐Term Cell Vitality and Osteogenic Induction of MSCs in LP Scaffolds

**Figure 3:**
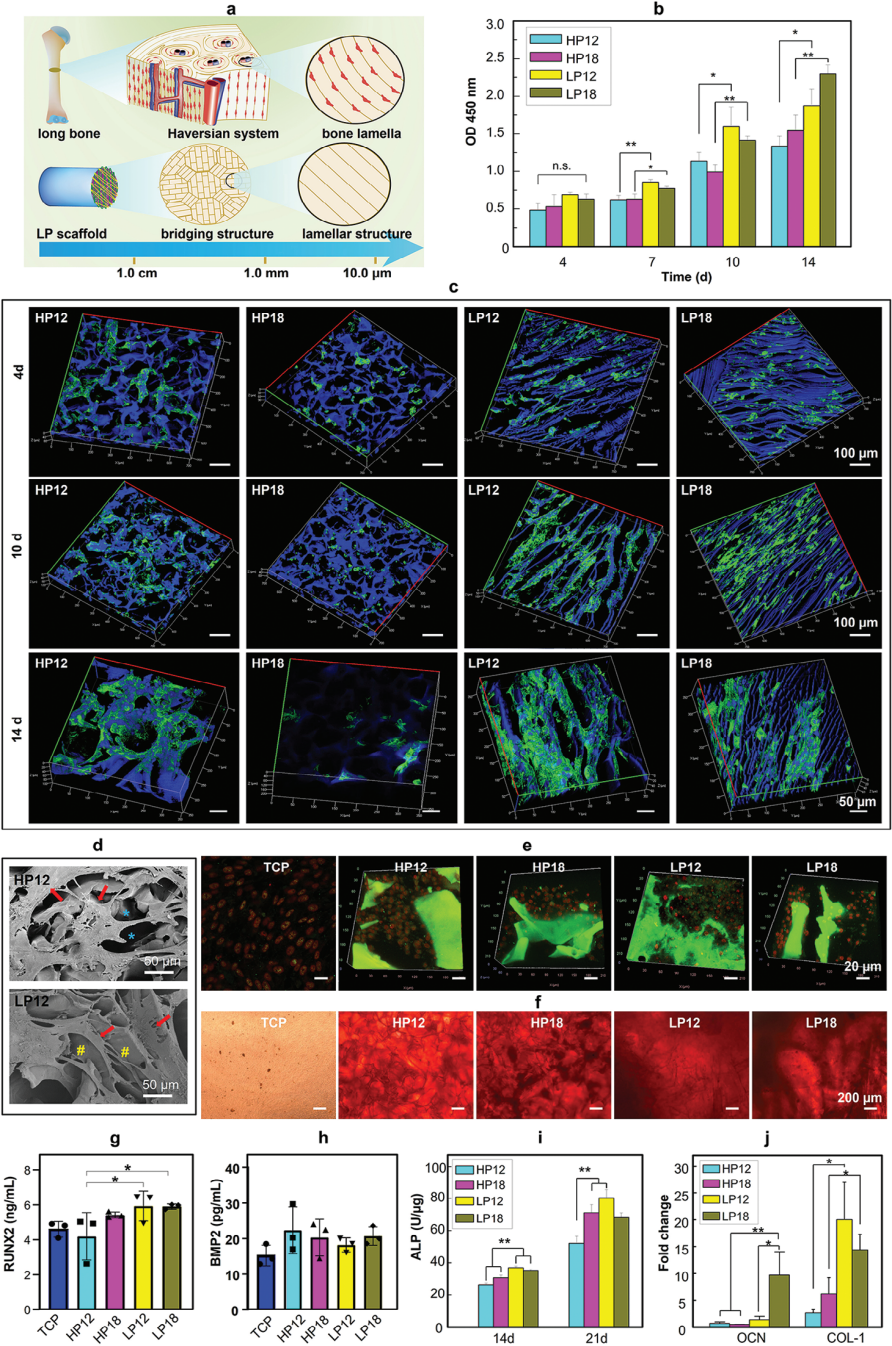
Morphology, proliferation, and osteogenic differentiation of MSCs in SF‐based 3D scaffolds. a) Schematic representation of the microscopic‐to‐macroscopic morphology of natural bone and LP scaffolds, showing that cortical bone tissue and LP scaffolds have similar lamellar structures at the micron level, which is conducive to cell adhesion and migration. B) Cell counting kit‐8 (CCK‐8) assay of long‐term cellular activity of hMSCs within SF‐based 3D scaffolds. c) The 3D confocal imaging of hMSCs within SF scaffolds after being cultured for 4, 10, and 14 d. Green fluorescence represents FITC‐labeled cells and blue fluorescence denotes the autofluorescence of SF scaffolds. d) SEM images showing that hMSCs on the lamella structures of LP scaffolds are clearly elongated along the lamellar extension direction, while hMSCs on the HP scaffolds are randomly elongated. Arrow: cell morphology; asterisk: hole‐wall of HP scaffolds; #: lamella wall of LP scaffolds. e,f) Immunofluorescence staining (e, BMP2 in cells is labeled green and RUNX2 in cells is labeled red; scaffolds also exhibit spontaneous green fluorescence in large areas.) and alizarin red staining (f) of rMSCs cultured in an osteogenic induction medium for 14 d. g,h) Quantitative analysis of RUNX2 (g) and BMP2 (h) concentrations by ELISA in the medium supernatant at 14 d. i,j) ALP quantitative analysis (i) and normalized OCN and collagen‐1 (COL‐1) mRNA levels (j) of hMSCs cultured in a normal medium for 14 d. (*n* = 3 for ALP and RT‐PCR). HP scaffolds: honeycomb porous scaffolds; LP scaffolds: lamellar porous scaffolds. Tissue culture plate (TCP) as the control group. All the data are presented as mean ± SD. ^*^ *p* < 0.05, ^**^ *p* < 0.01. n.s. denotes not significant difference.

In terms of the Haversian system of natural bone tissue, the obtained lamellar structures in the LP scaffolds were highly similar in morphology and scale to the natural bone lamella in vivo (**Figure** **3**a). Therefore, to evaluate the response of MSCs to lamellar structures, we seeded human MSCs (hMSCs) (Figure 3b–d) and rat MSCs (rMSCs) (Figure S3, Supporting Information) onto the scaffolds. Obviously, more hMSCs were found in the LP scaffolds than in the HP scaffolds (Figure 3b), and the cells spread better in the former after being cultured for 14 d (Figure 3c,d). Previous studies have shown that under 3D static cell culture conditions, seeded MSCs grow mainly on top of the porous 3D scaffolds rather than inside.^[^ ^26^ ^]^ To clarify this issue, we cut away the upper layer of the scaffolds at a height of ≈1 mm with rMSCs (Figure S3a, Supporting Information) and observed the survival of the cells inside the remaining scaffolds. Surprisingly, we found that the cells in the LP group were still evenly distributed inside the scaffold, while the cells inside the HP scaffolds were much less. These results demonstrated that the LP scaffolds have a favorable cellular affinity and can promote the migration and proliferation of MSCs around the lamellar structure.

To evaluate the effect of SF scaffolds on the differentiation of rMSCs, we seeded the rMSCs in an osteogenic induction medium for 14 d. The expression of key osteogenic markers, BMP2, and Runt‐related transcription factor 2 (RUNX2) was assessed through the immunofluorescence co‐staining (Figure 3e) and enzyme‐linked immunosorbent assay (ELISA, Figure 3g,h). Mineral deposits were visualized using alizarin red staining (Figure 3f). These results demonstrated that LP scaffolds (including LP12 and LP18) were more effective in inducing the differentiation of rMSCs toward osteogenic lineage when compared to the HP scaffolds in the osteogenic induction medium. To further substantiate these findings, we assessed the differentiation potential of hMSCs cultured without osteogenic differentiation inducers for both 14 d and 21 d. The results demonstrated that alkaline phosphatase (ALP) and osteocalcin (OCN) were significantly overexpressed in the LP groups compared with the HP groups, as evidenced by ALP activity (Figure 3i) and reverse transcription polymerase chain reaction (RT‐PCR) results (Figure 3j). Additionally, both LP12 and LP18 scaffolds presented positive signals for osteopontin (OPN) expression, whereas HP scaffolds showed nearly no expression (Figure S4, Supporting Information). Overall, the above results demonstrated that LP scaffolds, characterized by their lamellar structure, are more effective than HP scaffolds in promoting cell adhesion, proliferation, and osteogenic differentiation of MSCs. These characteristics are likely to facilitate cell recruitment and make LP scaffolds suitable for use as bone organoids in the regeneration and repair of bone defects.

**Figure 4:**
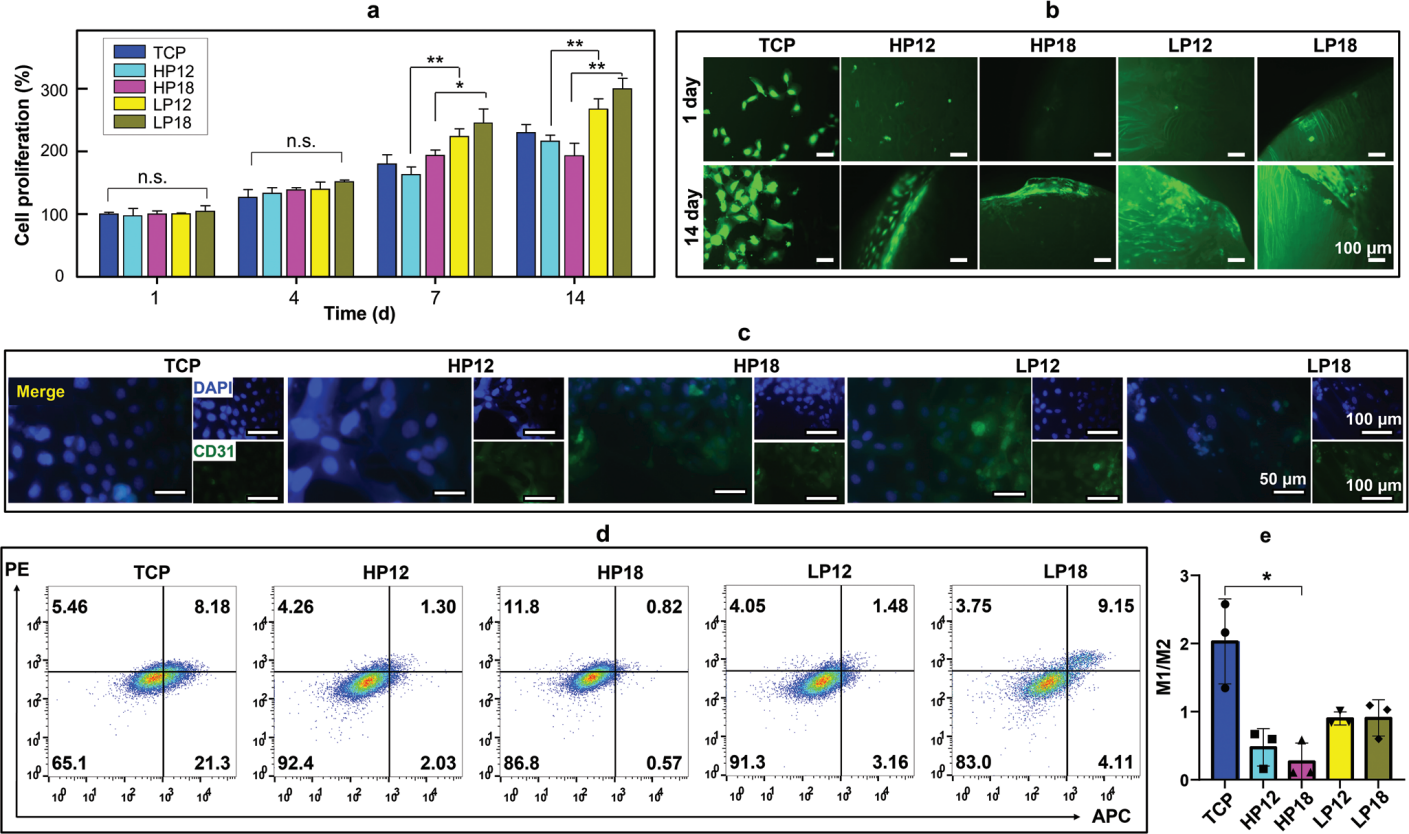
Effects of LP scaffolds on hUVEC cell behaviors and macrophage polarization. a) Proliferation assessment of hUVECs on LP scaffolds showing significantly enhanced proliferation compared to HP scaffolds. b) hUVECs morphology on the scaffolds. c) Immunofluorescence visualization of CD31 expression in hUVECs cultured on the scaffolds. d,e) Flow cytometry (d) and M1/M2 polarization graphs (e) of macrophages cultured on SF‐based scaffolds (LP and HP), highlighting a reduction in M1 polarization (CD86 expression) and an increase in M2 polarization (CD206 expression) relative to TCP. ^*^ *p* < 0.05, ^**^ *p* < 0.01. n.s. denotes not significant difference.

To elucidate the effects of LP scaffolds on vascularization and immune conditions in vitro, we cultured human umbilical vein endothelial cells (hUVECs, **Figure** **4**a,c) and RAW264.7 macrophages (Figure 4d,e) within the scaffolds for 3D cell culture. The cell proliferation assays demonstrated significant increases in hUVECs proliferation on the LP scaffolds at both 7 d and 14 d (Figure 4a). Additionally, the LP scaffolds exhibited favorable cellular affinity, promoting the migration of hUVECs along the lamellar structure (Figure 4b). Immunofluorescence analysis confirmed the expression of CD31, a well‐known marker of angiogenesis (Figure 4c), indicating the endothelial functionality of the hUVECs on LP scaffolds. To provide a comprehensive analysis of the immunomodulatory effects of the SF‐based scaffolds, we conducted flow cytometry to evaluate macrophage phenotypes (M1 and M2). The expression of pro‐inflammatory markers, such as CD86, and anti‐inflammatory markers, including CD206, were measured (Figure 4d,e). The quantitative results demonstrated that all SF‐based scaffolds, including both HP and LP scaffolds, had a lower potential for M1 polarization and a higher potential for M2 conversion compared to TCP. Collectively, these findings suggest that the LP scaffolds positively influence endothelial cell behavior and macrophage polarization, underscoring their potential for biomedical applications.

### 2.3 Effect of Bone Repair on Rat Bone Nonunion Model

**Figure 5:**
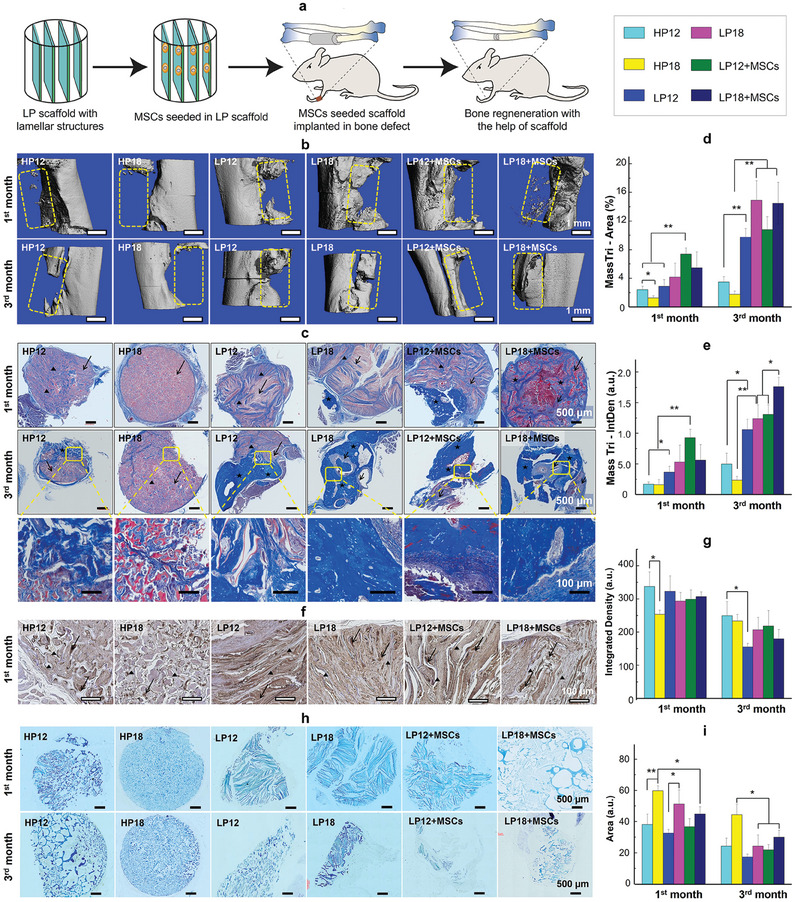
Repair of radial bone defects in rats using cylindrical LP scaffolds. a) Schematic illustration of LP scaffolds seeded with hMSCs to induce osteogenesis in a rat bone defect model. b) 3D micro‐CT images at 1 month and 3 months after scaffold implantation in the defect area. The dashed box shows the original defect area. c) Typical MT staining of the complexes of scaffolds and regenerated tissues after 1 and 3 months of implantation. Arrow: SF scaffolds; Triangle: newly regenerated bone tissue; Five‐pointed star: mature bone tissue. The bottom row of images shows magnified views of the MT staining in the third month. d,e) Semi‐quantitative results of area percentage (d) and intensity density (e) of MT staining indicate the area of regenerated bone and bone maturity, respectively. f,g) Immuno‐histological characterizations for OPN in scaffolds with or without hMSCs seeded after 1 month of surgery. Sections were counterstained with eosin after immuno‐histochemistry imaging (f). Arrow: OPN protein. Triangular: SF scaffolds. The integrated densities of OPN were quantified by the ImageJ software (g). h,i) Toluidine blue staining images (h) and semi‐quantitative results (i) indicating the degree of scaffold degradation in vivo. ^*^ *p* < 0.05, ^**^ *p* < 0.01.

We first constructed a rat bone nonunion model and then implanted the LP scaffolds into the defect models for 1 and 3 months to evaluate the bone repair effect of LP scaffolds in vivo (**Figure** **5**a). The nonunion gap between two ends of the bone defect was shortened and regenerative irregular minerals could be observed in the MSC‐seeded LP groups by Micro‐CT imaging after 1 month (Figure 5b), indicating that the new osteoid tissue began to grow at the MSC‐seeded LP groups at the initial stage of implantation. In the HP12 group, although a reduced area was observed, no signs of bone regeneration were found. However, the length of the bone defect remained almost unchanged in the HP18 group, indicating that honeycomb scaffolds could not promote early bone regeneration at high concentrations. After 3 months of implantation, we found that the bone defect was partially or completely bridged, which is a clear sign of bone healing in the LP12 and LP18 groups, either with or without hMSCs seeded. In contrast, less regenerative bone tissue was observed in the HP12 and HP18 groups than LP12 and LP18 groups (Figure 5b). Subsequently, Masson's trichrome (MT) staining (Figure 5c), and semiquantitative analysis (Figure 5d,e) were performed to analyze the distribution and maturity of the regenerated bone tissue in more detail. Pale‐blue (collagen fibers from fibrous tissue) and red (cytoplasm) staining were observed from MT staining in the LP groups at 1 month of implantation (Figure 5c), indicating that the host inflammatory cells and fibrocytes were recruited inside these scaffolds. Notably, the presence of a small piece of mineralized bone tissue (dark blue) was observed around the LP scaffolds after 1‐month implantation (Figure 5c,f). Among them, MSC‐seeded LP12 showed the most signs of new bone regeneration (Figure 5d,e).

To assess bone matrix expression, we utilized immuno‐histochemical staining with OPN (Figure 5f,g; Figure S5, Supporting Information) and OCN (Figure S6, Supporting Information) as markers for early and late stages of bone formation, respectively. All groups displayed stronger positive immunohistochemical staining for anti‐OPN antibodies in the 1st month compared to the 3rd month, indicating early expression of the bone matrix. After 3 months, we observed massive and intact calcified bone tissues (Figure 5c–e), along with marked OCN expression (Figure S6, Supporting Information) in all LP groups. This finding suggests that both LP scaffolds with or without MSCs effectively recruit host cells and promote bone tissue regeneration in rat bone nonunion models. Interestingly, we found that the LP18 group regenerated more bone tissues than the LP12 group, regardless of whether MSC was seeded (Figure 5d,e). These studies confirm that the LP12 scaffolds, due to their relatively loose pore structure, are more conducive to promoting bone regeneration in the early stages. While LP18 scaffolds, due to their high mechanical performance, are more favorable for promoting bone development in the late stage. The role of MSCs helps to increase the maturity of bone regeneration.

Moreover, scaffold biodegradation can provide suitable sites for regenerating bone tissue, and the scaffold degradation kinetics is highly related to the geometry of scaffolds.^[^ ^7a^ ^]^ Toluidine blue staining showed that compared with 1 month, the LP scaffolds and HP12 scaffolds were significantly degraded after 3 months of implantation, while the HP18 scaffold had the least degraded area (Figure 5h,i), indicating that a proper degradation rate of LP scaffolds can promote bone tissue regeneration. Therefore, the above results indicate that LP scaffolds with lamellar structure can recruit cells to penetrate the scaffolds in the early stage of implantation, thereby enabling scaffold degradation and bone remodeling.

### 2.4 Repair of LSBD in Bama (BM) Pigs with Large‐Size LP Scaffolds with Lamellae Perpendicular to the Bone Length Direction

**Figure 6:**
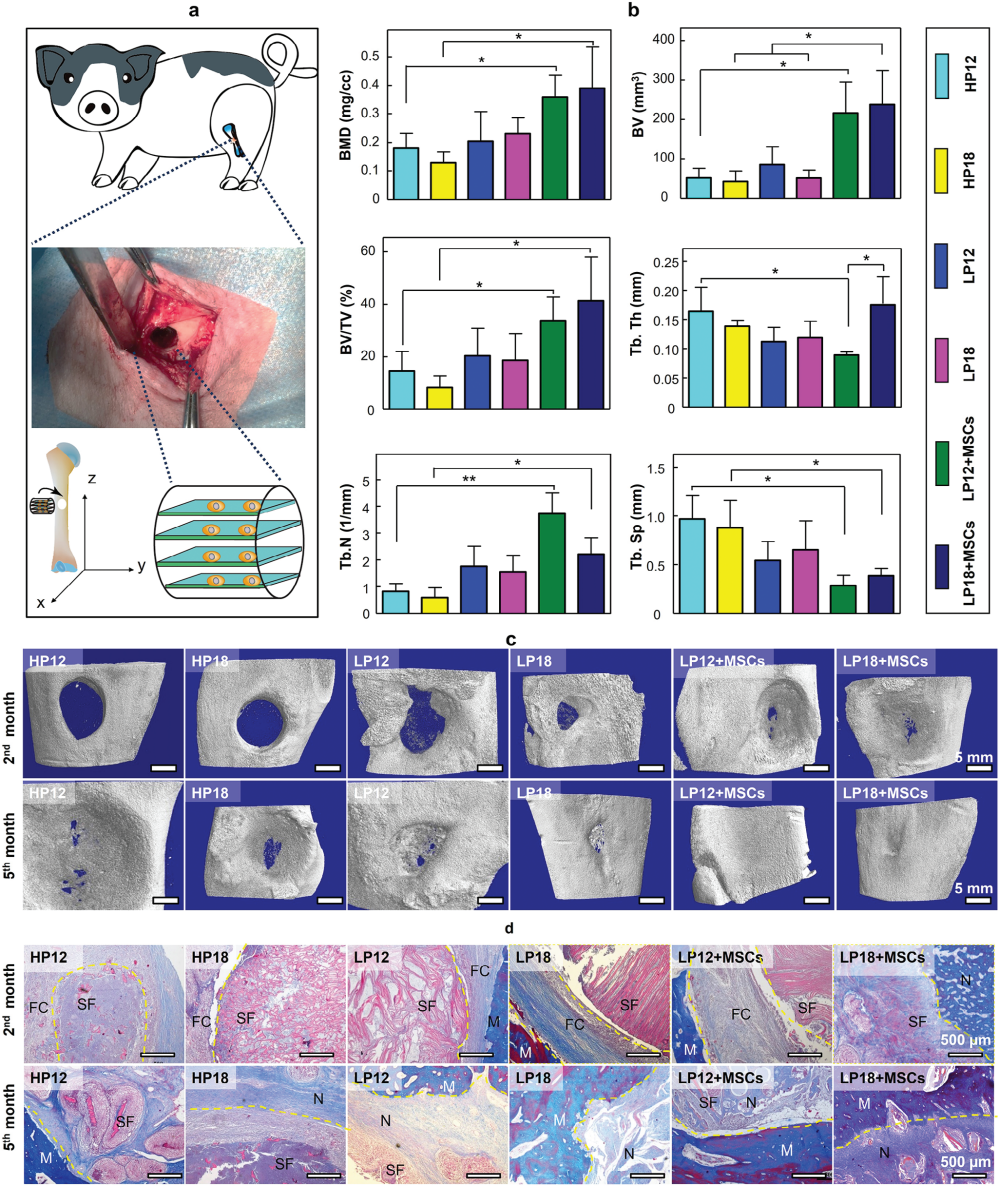
Bone regeneration analysis in pig tibial defects with LP scaffolds with lamellae perpendicular to the long axis (z‐axis) of the tibia. a) Schematic representation of the establishment of the pig LSBD model and the orientation and placement of the SF scaffolds at the defect site. b) Two‐month postoperative quantitative micro‐CT assessment of bone parameters, including BMD, BV, BV/TV, and trabecular bone attributes (Tb. Th, Tb. N, Tb. Sp). ^*^ *p* < 0.05, ^**^ *p* < 0.01. c) Representative micro‐CT images of pig tibial bone regeneration after 2 months and 5 months of implantation. d) Typical MT staining of scaffold‐tissue complexes was observed at 2 and 5 months after implantation. SF: SF Scaffold; N: Newly generated bone; M: Mature bone; FC: Fibrocytes. HP scaffolds: honeycomb porous scaffolds; LP scaffolds: lamellar porous scaffolds.

Although previous literature suggests that bone repair biomaterials hold great promising prospects for inducing in situ or ectopic bone formation in small animals,^[^ ^29^ ^]^ the use of SF‐based scaffolds to repair bone defects in large animal models remains challenging. Here, we implanted cylindrical LP scaffolds into the LSBD of the BM pigs with their lamellae perpendicular to the z‐axis (length direction) of the long bone (Vertical type implantation, **Figure** **6**a). Similar to the results of the rat model, the volume of LP scaffolds at the implant site was less than that of the HP18 group after 2 and 5 months of implantation (Figure S7, Supporting Information), indicating a faster in vivo biodegradation rate for LP scaffolds compared to the HP scaffolds. Representative 3D micro‐CT images revealed that the bone defect in the HP groups remained to be a circular cavity after 2 months, indicating the bone defect had not been healed (Figure 6c). In contrast, calcified callus appeared in the LP groups after 2 months, indicating that the circular bone defect was being repaired from the periphery toward the center. Importantly, the MSC‐seeded LP18 group showed the highest values for bone mineral density (BMD), bone tissue volume (BV), and the ratio of bone tissue volume to total tissue volume (BV/TV, Figure 6b), indicating an accelerated rate of bone tissue regeneration. After 5 months of implantation, we found that the LP12 and LP18 groups seeded with hMSCs successfully healed the bone defects completely, while there was still a small portion of unhealed tissue remaining in the LP12 group without hMSC‐seeded (Figure 6c). The HP18 group displayed the poorest healing outcome, with most defects still lacking signs of calcification (Figure 6b,c).

To explore the effect of the lamellar structure of the scaffold on the repair of LSBD, we observed the microstructure composition of the regenerated bone tissue by H&E staining (Figure S8, Supporting Information), and MT staining (Figure 6d). The findings from MT staining revealed no regenerated bone tissue was visible at the edges or center of the two HP scaffolds after 2 months, whereas new bone tissue traces were found at the bone defect edge of the LP groups (Figure 6d). After 5 months of implantation, a large area of regenerated bone tissue and some small degraded fragments of lamella structures were found in the LP18 groups with or without hMSCs being seeded. Moreover, we found that at the early stage of implantation (2 months), the bone defect in all LP groups was surrounded by fibrous tissue and a small number of granulosa cells (Figure S8, Supporting Information).

**Figure 7:**
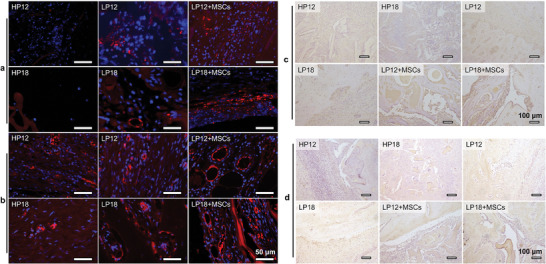
Evaluation of angiogenesis and bone matrix protein expression post‐scaffold implantation. a,b) Immunofluorescence staining for CD31 (red) for 2 months (a) and 5 months (b) post‐implantation with cell nuclei counterstained with DAPI (blue). c,d) Immuno‐histological characterizations for OPN (c) and OCN (d) in the BM pig bone defect model after 5 months. HP scaffolds: honeycomb porous scaffolds. LP scaffolds: lamellar porous scaffolds.

Angiogenesis is a crucial factor and can enhance bone repair in the healing of bone defects.^[^ ^9^, ^30^ ^]^ To verify whether the angiogenesis is related to the effect of bone repair, we further evaluated the vascularization in the newly formed bone/scaffold implantation site through immunofluorescence (**Figure** **7**a,b) and immunohistochemical staining (Figure S9, Supporting Information). We found that regenerated small‐diameter vessels were present in both the non‐seeded and hMSC‐seeded LP18 groups after 2 months. Surprisingly, the number and diameter of regenerated vessels in the LP18 groups with and without MSC seeding were both greater than those in the HP18 group without MSC seeding after 5 months of surgery. These results indicate that LP scaffolds can promote vascularization in the bone defect area compared to the HP scaffolds. The positive expressions of OPN and OCN were also observed in the LP groups after 2 months, among which the highest expression of OPN (Figure S10, Supporting Information) and OCN (Figure S11, Supporting Information) was observed in the hMSC‐seeded LP18 group. At the late stage of implantation (5 months), the LP groups exhibited a positive expression of OPN (Figure 7c) or OCN (Figure 7d), and the defect area was gradually replaced by new bone tissue. The above in vivo results in BM pig models were consistent with the results of rat bone defects, namely, the LP scaffolds with ordered lamellar structure could promote cell recruitment and proliferation, thus enhancing early vascularization, OPN expression, and the regeneration of the vascularized bone tissue at the LSBD site.

### 2.5 Repair of LSBD in BM Pig with Large‐Size LP Scaffolds with Lamellae Parallel to the Bone Length Direction

**Figure 8:**
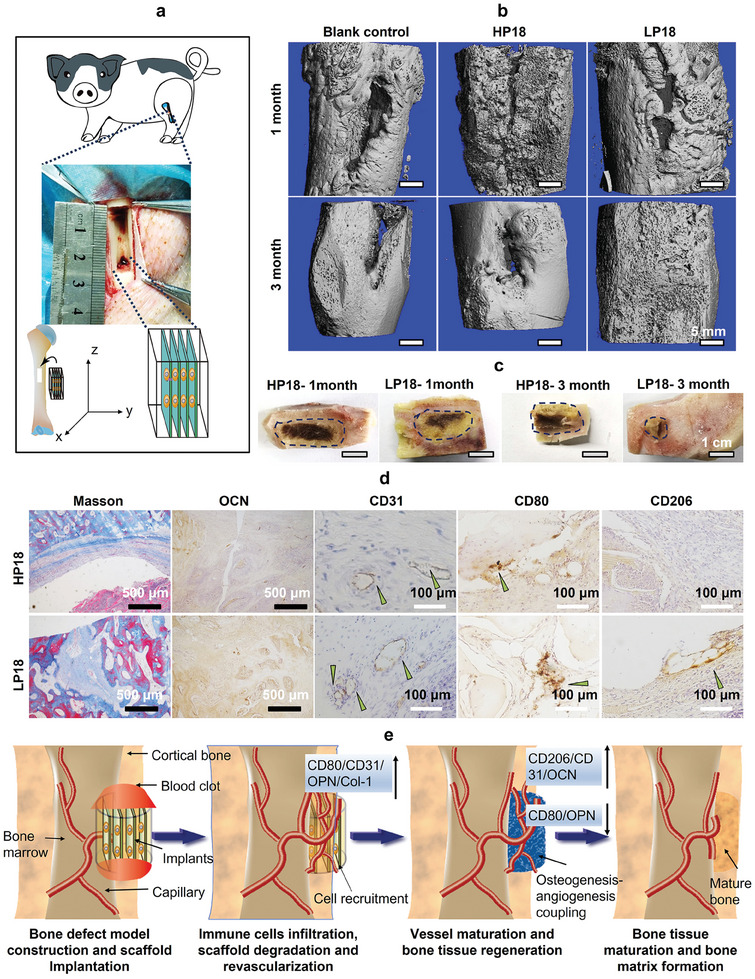
Vascularization and bone regeneration in pig defective tibia treated with LP scaffolds implanted with lamellae parallel to the z‐axis of the tibia. a) The schematic diagram shows the construction of the rectangular pig bone defect model and the implantation of rectangular SF scaffolds. b) Representative 3D micro‐CT images of regenerated pig bone at 1 and 3 months after implantation. c) Images show regenerated bone tissue after 1 month and 3 months. The dotted box indicates the location where the scaffold existed. d) MT staining and Immuno‐histological characterizations for OCN, CD31, CD80, and CD206 in pig bone defect model after 3 months of implantation. Arrows indicate the region where the corresponding antigen is positively expressed. e) Schematic representation of the process of LP scaffold‐induced vascularization‐based bone regeneration at the defective bone site. When the LP scaffolds are implanted, blood clots form around the LP scaffolds and attract inflammatory cells to recognize the scaffolds during the surgery. At the middle stage of implantation, the LP scaffold degrades in an orderly manner under the influence of macrophages in vivo; at the same time, small vessels and calluses gradually form at the site of implanted scaffolds. In the later stage, the LP scaffold is degraded in vivo, and the callus tissue transforms into structurally ordered mature bone tissue.

To verify the effect of the lamellar structure implantation direction on the bone regeneration effect, we prepared a large‐size cuboid LP scaffold with a length, width, and height of 2.5 × 1.0 cm × 0.6 cm to repair the BM pig defect bone tissue in the direction of lamella parallel to the z‐axis of the bone (Parallel type implantation, **Figure** **8**a). Since LP18 scaffolds have shown the best effect on bone tissue regeneration, we directly compared LP18 and HP18 to observe the effect of the orientation of the microlamellar structure on bone tissue regeneration. After 1 month of implantation, a noticeable cuboid gap was observed within the control group, while a small gap was evident on the outer surface of the defective bone in the LP group (Figure 8b). Besides, marks of the gap were still visible in the HP group after 3 months of implantation, indicating the bone defect had not been repaired by the HP scaffolds. However, 3D micro‐CT results (Figure 8b) and macroscopic observation (Figure 8c) showed that the bone defect site had healed in the LP groups. Thus, the LP groups showed a better effect in promoting bone regeneration than the control groups, and the parallel type implantation was more effective in bone repair than the vertical type implantation experiment in BM pigs after 5 months (Figure 6c). Therefore, these results showed that LP scaffolds had the optimal effect of promoting bone regeneration, and the implantation direction of lamellar scaffolds could affect the bone regeneration rate.

The main influencing factors of bone regeneration are not only closely related to the rate of scaffold degradation and angiogenesis but also related to the micro‐environment of osteoimmunomodulation.^[^ ^9^, ^31^ ^]^ Therefore, the role of inflammatory cells and angiogenesis in bone tissue regeneration was analyzed by immunohistochemistry and MT staining (Figure 8d; Figure S12, Supporting Information). CD80 was selected as a marker of M1‐type macrophages at the initial stage of scaffold implantation to demonstrate the inflammatory state and the foreign body removal level at the defect site. Also, CD206 was selected as a marker for M2‐type macrophages to indicate the level of anti‐inflammation and wound healing. The positive expressions of CD80 were observed in all groups after 1 month of implantation, while the positive expressions of CD206 were observed only in the LP groups (Figure S12, Supporting Information). After 3 months, the positive expressions of CD80 and CD206 were still observed in the LP groups (Figure 8d), confirming that the lamellar structure in the LP scaffolds can promote the polarization of macrophages from the M1 phenotype into the M2 phenotype.

Afterward, to verify whether macrophage polarization affects the angiogenesis and the expression of bone matrix protein, we used the CD31 antibody to label the regenerated blood vessels and the OCN antibody to label the regenerated bone tissue. Positive expression of CD31 and abundant expression of OCN were found in the LP scaffolds after 1 month (Figure S12, Supporting Information). Notably, OCN proteins and larger diameter vessels were found in the LP groups at a later stage (after 3 months), suggesting that the polarization of macrophages can regulate angiogenesis and bone matrix deposition (Figure 8d). It is worth noting that MT staining showed that the LP18 scaffolds almost completely degraded and formed a cancellous bone 3 months post‐implantation (Figure 8d). The newborn trabeculae in callus increased gradually and the arrangement tended to be regular and dense. This observation directly indicated that the regenerated bone tissue was gradually integrated into the bone reconstruction process, forming mature bone tissue. These results indicated that LP scaffolds implanted with their lamellae parallel to the long‐axis orientation of long bone could rapidly recruit body cells, which in turn accelerate bone matrix formation and calcified bone maturation by modulating macrophage status and vascularization (Figure 8e).

## 3 Discussion

The effective treatment of nonunion injuries and LSBD remains a major challenge in bone tissue engineering research and orthopedic surgery. The embarrassing situation is that most studies on bone defect repair often focus on the addition of exogenous growth factors to endow scaffolds with osteoinductivity and promote bone regeneration.^[^ ^6^, ^9^, ^17^, ^32^ ^]^ However, the ability of scaffolds to recruit host cells and endogenous growth factors in vivo is often neglected. The process of treating bone defects through bone tissue engineering therapy is essentially a carefully orchestrated series of biological responses between tissue and scaffolds in vivo. Here, we prepared a bone‐like scaffold with a homogeneous lamellar structure, good pore connectivity, and excellent mechanical properties by a freeze‐casting method (Figure 2). The micro‐topological structure of the obtained lamellar scaffold not only resembles the bone laminae of cortical bone in shape and scale but also exhibits improved mechanical strength and cell affinity compared to honeycomb scaffolds. This structure supports cell colonization, migration, proliferation, and differentiation around the lamellae.

During freeze‐casting, the scaffolds undergo directional solidification through ice crystal templating, which imparts a well‐defined orientation to the material along the z‐axis, providing enhanced mechanical support and increasing compressive strength and Young's modulus (Figure 2g,h). In contrast, the freeze‐drying method typically occurs in a more random porous structure, which exhibits isotropy and is more susceptible to deformation under compression along the z‐axis, leading to lower compressive strength and Young's modulus. Additionally, in freeze‐casting, increasing the distance from the cold end results in thicker lamellae within SF frameworks, leading to a reduction in porosity along the z‐axis and a denser interlocking between the lamellae.^[^ ^20^, ^33^ ^]^ Furthermore, in this study, freeze‐casting can optimize mechanical properties by designing gradient laminar structures. By adjusting parameters such as freezing rate, slurry composition, and concentration (Figure 2a,b), the thickness of the laminar layers can be altered to further enhance compressive strength and modulus (Figure 2e–h).

The LP scaffolds exhibit higher hydrophilicity than the HP scaffolds due to their ordered and continuous pore arrangement,^[^ ^34^ ^]^ which not only facilitates the permeation of bodily fluids and cytokines but also accelerates the diffusion of degradation media, providing an ideal environment for scaffold degradation in vivo. In contrast, the HP scaffolds have a more closed and random pore structure, restricting the permeation and diffusion of bodily fluids, and thereby slowing down the degradation rate (Figure 2a,d). Moreover, the ordered pores of the lamellar structured scaffolds facilitate the timely removal of degradation products, which is beneficial for maintaining the continuous progression of the degradation process (Figure 2d). In contrast, HP scaffolds hinder the effective recruitment and activation of endogenous cells and affect the clearance of degradation products, thus reducing degradation efficiency. Furthermore, we observed that after seeding MSCs onto the LP scaffolds, cells can form a 3D network within the scaffold (Figure 3c,d), facilitating cell‐to‐cell interaction and signal transduction, which helps enhance the degradation capability of LP scaffolds (Figure 5h,i).

Additionally, the LP scaffolds, by providing more surface area and space, are conducive to the recruitment, adhesion, proliferation, and differentiation of endogenous cells, such as macrophages, osteoprogenitor cells, and vascular endothelial cells (Figures 7a, 8d; Figure S12, Supporting Information). The increased activity and number of cells contribute to the secretion of enzymes and other bioactive molecules, promoting the regeneration of new bone tissue.

To prove the effectiveness of the LSBD repair ability of the lamellar scaffold in repairing bone defects in vivo, we constructed two animal bone defect models (rat bone nonunion model and BM pig large bone defect model) for validation. In the rat bone nonunion model, we found that the LP scaffolds promoted the regeneration of the bone tissue, healing the defects within 3 months (Figure 5b–e). The LP scaffolds were also gradually biodegraded during this healing process (Figure 5h,i). For the BM pig model, we found the LP scaffold‐based tissue regeneration process can be roughly divided into 3 stages. During the early stage and the middle stage, immune cell infiltration (Figure 8d), angiogenesis (Figures 7a, 6b and 8d), and scaffold degradation (Figure 8c; Figure S12, Supporting Information) play different roles. In this regard, immune cell infiltration initiates the inflammatory response, providing necessary conditions for the growth of new tissue. Vascularization plays a key role in early bone remodeling,^[^ ^35^ ^]^ enabling early mineralized callus to accumulate rapidly at the periphery of the scaffold. Meanwhile, scaffold degradation offers space for the growth of new tissue, maintaining a hypoxic state in the implantation site, which is conducive to promoting tissue regeneration.^[^ ^36^ ^]^ In the later stage of repair, callus gradually evolved into the mature and well‐ordered mineralized bone under the dual action of osteoclasts and osteoblasts, which eventually regenerate into the original structure of bone tissue (Figures 6b–d and 8b–e).

Despite the considerable advancement achieved in the development of SF‐based LP scaffolds prepared by a freeze‐casting method for repairing large segmental bone defects in our work, limitations remain, and addressing these limitations constitutes instances of future orientations. Our research confirms the excellent bone regeneration performances of the LP scaffold system, but due to the complexity of the system, it is not possible to independently regulate the material properties of the LP scaffold (such as surface area and mechanical strength) and thus determine the source of this excellent performance. In fact, the majority of the present studies regarding the scaffold for bone regeneration concentrate on an individual factor, either biochemical or biophysical stimulation for determining the performance of repairing bone defects.^[^ ^37^ ^]^ An ideal scaffold system is needed to untangle the impacts of different material cues on the fate of bone regeneration independently. In addition to using SF, which is a nature‐derived biomacromolecule, inorganic material systems such as biomimetic hydroxyapatites have also been used to prepare 3D scaffolds as bone graft substitutes recently.^[^ ^16d^ ^]^ More systematic cross‐comparative researches are necessary to determine which material system is more suitable for preparing freeze‐casting scaffolds for bone regeneration.

## 4 Conclusion

We constructed for the first time a bone repair scaffold with a homogeneous lamellar structure based on SF protein by freeze‐casting. The resultant scaffold has a topography similar to the microstructure of cortical bone at the micrometer scale. Compared to the honeycomb scaffolds, the lamellar scaffolds had better mechanical strength and faster degradation rate, which can promote cell adhesion, maintain long‐term cell vitality, and induce better osteogenesis. More interestingly, the lamellar scaffolds implanted with lamellae parallel to the long axis direction of diaphysis can effectively recruit inflammatory cells, endothelial cells, and fibroblasts in the rat bone nonunion model and the BM pig critical‐sized bone defect model, promoting the neo‐vascularization as well as degradation at an appropriate rate to eventually form mature bone tissue in situ. These results provide a good precedent for repairing bone tissue without the addition of growth factors, simply by changing the microstructure of the scaffolds and the implantation method. Therefore, the SF lamellar scaffolds based on freeze‐casting technology provide a potential avenue for the development of cortical bone organoids and bone repair strategies.

## 5 Experimental Section

### Preparation of SF Solution

SF solution preparation starts with the degumming of silk cocoon fragments in Na_2_CO_3_ aqueous solution and then dissolving them in a 9.3 m LiBr aqueous solution as outlined in a previously described procedure.^[^ ^24^, ^38^ ^]^

### Preparation of Honeycomb‐Like Porous (HP) Scaffolds

The SF solution with a concentration of 6% (w/v), 12% (w/v), 18% (w/v), or 22% (w/v) was poured into a cylindrical or cubic plastic mold with an inner diameter of 1 cm, respectively, then stored overnight at −20 °C in a refrigerator. After freezing, frozen SF was freeze‐dried by using a freeze dryer (FD‐1D‐50, Biocool, China) for 36 h to form a 3D scaffold with a honeycomb‐like porous structure. Methanol treatment was performed by soaking honeycomb‐like porous scaffolds in 80% methanol aqueous solution for 12 h to make the scaffolds insoluble.

### Preparation of Lamellar‐Like Porous (LP) Scaffolds

The SF solution with the concentration of 6% (w/v), 12% (w/v), 18% (w/v), or 22% (w/v) was poured into a cylindrical mold or cubic with an inner diameter of 1 cm, respectively. The resultant LP scaffolds were made by freeze‐casting of these suspensions and thus termed LP6, LP12, LP18, and LP22, respectively. Specifically, a freeze‐casting device designed in the laboratory consists of a copper rod, a heater coil, a liquid nitrogen storage box, and a temperature control system that was used to cast LP scaffolds. The outside of the copper rod is wrapped by a heat‐conducting coil, and the top is used to place the mold, while the bottom is immersed in a liquid nitrogen box. The cooling rate of the mold is precisely controlled by the temperature control system. The operating conditions of freeze‐casting used were as follows: the range of cooling temperature was reduced from 5 to −140 °C at a constant freezing rate of 3 °C min^−1^. After the process, the mold with solid SF/ice crystals was removed and placed in a freezing dryer for 36 h to obtain lamellar structured LP scaffolds. Similarly, to make the LP scaffolds insoluble in water, were soaked them in an 80% methanol solution for 12 h.

### Characterization of Porous Scaffolds

For SEM observation: The cross‐section and longitudinal profile of the 3D porous scaffolds were sputtered with gold, and the scaffolds underwent observation and imaging by using SEM (SU‐8010, Hitachi). The lamella spacing and lamella thickness of lamellar‐like porous scaffolds were calculated by the NIH‐ImageJ software. For quantification analysis, each group was repeated 50 times and analyzed statistically. To observe the internal topography of the LP scaffold, the was embedded scaffold in paraffin and sliced it with a slicer to obtain 6 µm thin slices, which were observed and imaged by a light microscope. For mechanical properties: SF scaffolds with a diameter of 1.0 cm and a height of 1.5 cm were measured by a universal mechanical tester (AGS‐J, Shimadzu) equipped with a 50 N load cell under ambient temperature. The stress–strain profiles of the scaffolds were recorded, and the compressive modulus was derived from the gradient of the initial linear‐elastic phase of the stress–strain curve.

### In Vitro Degradation Analysis of Scaffolds

To determine the degradation of scaffolds under different states, the PBS, protease from *Streptomyces griseus* (P8811‐1G, Sigma–Aldrich, Co.) with enzyme activity of 1 and 10 U mL^−1^ were set up at 37 °C. Meanwhile, to simulate degradation under exercise, these groups were placed in a shaker shaken at a rate of 180 rpm. Three replicates were done for each group, and the mass loss rate of the scaffolds was determined every 5 days.

### The Proliferation of Cells

To evaluate cell adhesion and proliferation of cells on the scaffolds, hMSCs (Bone‐derived, ATCC) and rMSCs (RASMX‐01001, Cyagen Biosciences) were employed. SF scaffolds were immersed in 75% alcohol for 12 h, washed 3 times in PBS for 6 h each time, and soaked in cell medium twice for 12 h each time before cell seeding. 100 µL of suspended MSCs at a concentration of 5 × 10^4^ cells well^−1^ was seeded into the scaffolds. After half an hour, 400 µL of cell medium was added to each well. Due to the collapse of the internal lamellar structure of the 6% SF scaffolds after ethanol treatment, it is not suitable for cell and subsequent experiments. Similarly, the 22% LP scaffold also has an irregular lamellar structure, so it is not used. Cell proliferation was analyzed using the CCK‐8 assay kit (Biosharp) to assess cell proliferation and scaffold compatibility. After MSCs were cultured for 4, 7, 10, and 14 d, CCK‐8 reagent was introduced to each specimen and incubated at 37 °C for 2.5 h. The absorbance, indicated by the optical density (OD), was recorded at 450 nm by a microplate reader (Bio‐Rad 680, USA).

### Morphology of Cells

SEM observation of cells/scaffolds: SEM observation for cells/scaffold complex was conducted. Briefly, the complexes were washed with PBS, fixed in a 4% glutaraldehyde solution for 12 h, dehydrated with an ethanol solution, and dried in a critical point dryer. The cell/scaffold surface characteristics were observed under a SEM (TM‐3000, Hitachi). Confocal laser scanning microscopy (CLSM) observation of the MSCs on the top of scaffolds: hMSCs or rMSCs after 4 d and 10 d culture were rinsed with PBS and then fixed with a 4% glutaraldehyde solution. The cytoskeletons of MSCs were stained by the green fluorescent dye (Alexa Fluor 488 dye, Thermo Fisher Scientific) and observed by CLSM (LSM780, ZEISS). For observing MSCs inside the LP scaffolds: the fixation and staining methods of MSCs were the same as those of MSCs on the top of scaffolds. After that, a sharp blade was used to remove the upper layer of the scaffolds by ≈1 mm, enabling the observation of MSC morphology within the scaffolds.

### Evaluation of Osteogenic Differentiation of rMSCs in Osteogenic Induction Medium

Preparation of osteogenic induction medium: standard rMSCs medium (RAXMX‐90011, Cyagen Biosciences) supplementation with 50 µg mL^−1^ ascorbic acid (A103539, Aladdin), 10 mM β‐ Glycerol phosphate disodium salt (G806967, Macklin) and 10 nM dexamethasone (A17590, Alfa Aesar). The osteogenic differentiation efficiency of MSCs in osteogenic induction medium was examined by ELISA, alizarin red, and immunofluorescent staining. Scaffolds (HP12, HP18, LP12 and LP18) were placed in 24‐well plates, with 5×10⁵ MSC cells incubated on top. The cell samples were incubated for 14 d. For immunofluorescence staining: The cells digested from the scaffold using trypsin were initially subjected to incubation with primary antibodies specific for BMP2 (mouse monoclonal, Ag13501, Proteintech) and RUNX2 (rabbit monoclonal, AF2593, Beyotime), followed by incubation with DyLight 488 Goat anti‐mouse IgG (A23610, Abbkine) and DyLight 649 Goat anti‐rabbit IgG (A23620, Abbkine). The high‐resolution laser confocal microscope (LSM 880, Zeiss) in 3D imaging mode was used to observe the expression of BMP2 (green) and RUNX2 (red). For alizarin red staining, the cell samples were stained with alizarin red (G1450, Solarbio Life Science) and observed under light microscopy (IX71, Olympus). For ELISA, the cell samples were incubated for 14 days and then the cell supernatants were collected, and the concentrations of BMP2 and RUNX2 were determined by Rat RUNX2 ELISA Kit (JL43515, Jonlnbio) and Rat BMP2 ELISA Kit (JL11722, Jonlnbio), respectively.

### Evaluation of Osteogenic Differentiation of hMSCs in Standard MSC Medium

Following culturing for 14 and 21 d, the osteogenic differentiation efficiency of MSCs in these scaffolds with *standard MSC medium* (HUXMA‐90011, Cyagen Biosciences) was examined by ALP, RT‐PCR, and immunofluorescent staining. For ALP analysis: ALP activity from cell lysates was detected using an alkaline phosphatase chromogenic assay kit (Beyotime Biotechnology, China). To calculate the absolute content of ALP, the ALP was quantitatively expressed by standard BSA protein concentration. The mRNA expressions of OCN and COL‐1 were detected by RT‐PCR. The total RNA of MSCs was extracted by a Cells‐to‐CDNA II Kit (Thermo Fisher Scientific), followed by cDNA synthesis using the PowerUp SYBR Green Master Mix kit (Thermo Fisher Scientific). The quantification of target mRNA expression levels was performed using the 2^−△△Ct^ method with normalization to the reference gene of GAPDH (**Table** **1**). For immunocytochemistry imaging: After 21 d of culture, hMSCs were harvested and fixed in 4% paraformaldehyde solution. hMSCs were then incubated with the primary anti‐osteopontin antibody (ab8448, Abcam) overnight, the Alexa Fluor 594 ‐conjugated secondary anti‐rabbit antibody (ab150080, Abcam), and green fluorescent dye (Alexa Fluor 488 dye, Thermo Fisher Scientific) before observed by a laser confocal microscopy (LSM780, ZEISS).

**Table 1:** Primer sequences used for RT‐PCR.

Genes	5′‐3′	Primers
GAPDH	Forward	TGACGCTGGGGCTGGCATTG
	Reverse	GGCTGGTGGTCCAGGGGTCT
OCN	Forward	AGGGCAGCGAGGTAGTGAAGA
	Reverse	TAGACCGGGCCGTAGAAGC
COL‐1	Forward	ATGGATTCCAGTTCGAGTAGGC
	Reverse	CATCGACAGTGACGCTGTAGG

### Evaluation of Endothelialization of hUVECs

hUVECs (C2519A, Lonza) were seed in endothelial cell growth medium (EGM‐2, CC‐3162, Lonza) at a density of 1×10^5^ cells cm^−^^2^. The cells were cultured in an environment at 37 °C with 5% CO_2_ until they reached 80–90% confluence. Following this, 400 µL of the hUVECs suspension was added to each scaffold in a 24‐well plate. For cell proliferation assay, MTT assays were performed at 1, 4, 7, and 14 d post‐seeding according to the manufacturer's instructions using the MTT cell growth assay kit (M6494, Thermo Fisher Scientific Inc.). For morphology observation of hUVECs, the cells were stained with calcein‐AM (C3099, Thermo Fisher Scientific Inc.) staining for 40 min at 37 °C, which allowed for the visualization of live cells. For Immunofluorescence staining: After 14 d of cell culturing, scaffolds were fixed with 4% paraformaldehyde for 15 min, followed by incubating with primary antibodies (CD31, ab28364, Abcam) overnight at 4 °C, followed by incubating with secondary antibodies conjugated to Alexa Fluor 488 (ab150077, Abcam), followed by staining with DAPI for another 10 min and visualize under a fluorescence microscope.

### Evaluation of Macrophage Polarization

TCP and four types of scaffolds (HP12, HP18, LP12, LP18) were placed in 24‐well plates and inoculated with 5×10^5^ RAW264.7 cells (M3‐0101, Cyagen Biosciences). Following a 48 h incubation period, cell samples were collected by centrifugation and stained for APC anti‐mouse CD86 (20‐0862, Tonbo Biosciences) and PE anti‐mouse CD206 (E‐AB‐F1135D, Elabscience). Subsequently, each sample was transferred to a flow tube, where M1 and M2 macrophages were identified through flow cytometry (FACSVerse, BD Biosciences).

### Construction of Rat Defect Bone Model and Scaffold Implantation

Evidence suggests that MSCs derived from various sources may show lower immunogenicity than other cell types, making them suitable candidates for applications in regenerative medicine.^[^ ^10^, ^39^ ^]^ In here, Sprague‐Dawley (SD) rats were assigned to six experimental groups (HP12, HP18, LP12, LP18, LP12 scaffold seeded with hMSCs, and LP18 scaffold seeded with hMSCs) along with one blank control group. Each group included 4 rats for subsequent experimental. Briefly, anesthesia was administered via 4% isoflurane/oxygen inhalation using a VT‐110 small animal anesthesia machine. A 5 mm gap was created in the middle part of the radius using a surgical scissor. Meanwhile, the periosteum of the bone defect was removed. Following hemostasis, a 5 mm‐long and 3 mm‐diameter SF scaffold was surgically placed into the defect site. The incision was sutured, and Lidocaine (1 mL kg^−1^) was injected into the surgical area. The blank control group performed the same procedure as the experimental groups, excluding scaffold implantation. After surgery, each rat was subcutaneously injected with the ketotifen solution with the dosage of 1 mL kg^−1^ every 24 h for 3 d to prevent infection. After implantation for 1 month and 3 months, rats were euthanized and the implants along with the forelimbs were retrieved, fixed in 10% neutral formalin solution, and prepared for subsequent analysis.

### Evaluation of Rat Bone Regeneration: Mico‐CT Analysis

The samples were positioned in a cylindrical tube and scanned with a high‐resolution mico‐CT system (Scanco Medical, Switzerland), with a resolution of 10.5 µm. The resulting data were utilized to generate detailed 3D reconstructions, enabling visualization of the degree of calcified tissue. Following micro‐CT analysis, the samples were decalcified using EDTA decalcifying solution (D0818, Sigma) on a shaking table for 34 d. Subsequently, the decalcified implants were embedded in paraffin by a paraffin embedding station (EG 1160, Leica) and sectioned into slices ≈5 µm thick using a paraffin microtome (RM 2155, Leica). Prior to staining, the slides were thoroughly rinsed in PBS and incubated in a hydrogen peroxide block for 10–15 min. Staining procedures: MT staining was performed to verify the formation of new bone formation, while Toluidine blue staining was used to verify the degradation of SF scaffolds. For immunohistochemical staining, these tissue slices were subjected to immunohistochemical staining by incubation with primary antibodies specific to osteocalcin (sc‐30044, Santa Cruz Biotechnology) and osteopontin (sc‐21742, Santa Cruz Biotechnology) to evaluate bone formation markers. HRP‐conjugated secondary antibodies and diaminobenzidine (DAB) were employed for staining, followed by counterstaining of nuclei with hematoxylin to enhance contrast. To quantify the amount of bone tissue from immunohistochemical staining images, all images were captured at a consistent magnification, and 20 random sections from each group were selected for analysis. Chromogen intensity and area were quantified using ImageJ software with the IHC Profiler plugin, enabling precise quantification of staining outcomes and facilitating comparative analysis.

### Construction of BM Pig Bone Defect Models and LP Scaffolds Implanted Vertically to the z‐Axis of the Tibia

Construction of pig bone defect model: 6‐month‐old BM pigs weighing ≈25 kg were selected and divided into 7 groups for this experiment. The pigs were anesthetized by ear intravenous injection of sodium pentobarbital (25 mg mL^−1^, 8 mL), then securely positioned on the operating table after shaving and disinfection. The procedure involved cutting the skin of the lower limbs, dissecting the muscles, exposing the tibia, and removing the periosteum from the bone's outer surface. Subsequently, two cylindrical notches, each with a depth of 1.1 cm and a diameter of 1.0 cm were created by a medical bone drill in each pig's two tibia bones. Before implantation, hMSC‐seeded scaffolds were prepared by seeding hMSCs on LP scaffolds and incubating for 7 d. Then the prepared scaffolds, with or without hMSCs, measuring 1.0 cm in length and 1.0 cm in diameter were implanted into the bone defect site. Following implantation, the muscle and skin were sutured respectively, and 1.5 mL of 2% lidocaine was injected subcutaneously. The animals' wound healing, vitality, and infection status were regularly observed. After 2 months and 5 months post‐surgery following the guidelines of the Zhejiang University Laboratory Animal Ethics Committee. Subsequently, the bone tissues were extracted and fixed in a 10% neutral formalin solution for further analysis.

### Evaluation of Bone Regeneration of BM Pigs

Initially, the scaffolds along with bone tissues were scanned with a Micro‐CT system (Skyscan 1076, Bruker) to obtain high‐resolution images for further analysis. Then, the samples were decalcified using 12 wt.% EDTA decalcifying solution for 5 months to prepare them for subsequent histological examinations. Subsequently, the implants were paraffin‐embedded, sectioned, and subjected to MT staining for the verification of new bone formation. Immunohistochemical staining was carried out by incubating the tissue slices with primary antibodies targeting key markers of bone formation, including osteocalcin (ab13420, Abcam), osteopontin (ab8448, Abcam) and CD31(ab28364, Abcam). The staining process involved the use of an HRP‐conjugated secondary antibody and diaminobenzidine (DAB) to visualize the expression of these markers, with nuclei counterstained using hematoxylin. Furthermore, immunofluorescent staining was performed using a primary antibody against CD31(ab28364, Abcam), followed by incubation with a fluorochrome‐conjugated secondary antibody, and DAPI for nuclear staining.

### Construction of BM Pig Critical‐Sized Bone Defect Models and LP Scaffolds Implanted with Lamella Perpendicular to the z‐Axis of the Tibia

The procedure for constructing the critical‐sized bone defect model closely mirrored that of the BM bone defect model, with the key distinction being the creation of a cube‐shaped incision on the tibia measuring 2.5 × 1.0 cm × 0.5 cm. Scaffold implantation: Prior to implantation, hMSC‐seeded scaffolds were prepared by culturing hMSCs on the LP scaffolds for 7 d. Subsequently, scaffolds, whether seeded with hMSCs or not, sized 2.5 cm in length, 1.0 cm in width, and 0.5 cm in height were implanted into the bone defect site. Following 1 month and 3 months post‐surgery, the animals were humanely euthanized and the scaffolds along with bone tissues were carefully extracted and preserved in a 10% neutral formalin solution for further analysis. Throughout the experimental timeline, the animals' well‐being and recovery progress were closely monitored in adherence to the guidelines established by the Zhejiang University Laboratory Animal Ethical Committee (No. ZJU20230236) and the Laboratory Animal Ethical Committee of Jiangxi Zhonghong Boyuan Technology Co., Ltd. (No. 2020122501).

### The Role of Immune Cells and Angiogenesis

The scaffolds along with bone tissues were scanned with a Micro‐CT system. Then, the samples were decalcified using 12wt.% EDTA decalcifying solution for 5 months. The decalcified implants were embedded in paraffin and then sectioned into slices. For MT staining: an MT staining kit was used to verify the formation of new bone formation. For immunohistochemical staining, these tissue slices were immunostained using primary antibodies specific to osteocalcin (ab13420, Abcam), the primary antibody against osteopontin (ab8448, Abcam), (CD206, Novus Biologicals) and the primary antibody against CD31(ab28364, Abcam). HRP‐conjugated secondary antibody and diaminobenzidine (DAB) were used to stain these sections, and then nuclei were counterstained with hematoxylin.

### Statistical Analysis

Data analysis utilized an unpaired two‐tailed t‐test through GraphPad Prism 5. The sample size was n ≥ 3 for statistical analysis of in vitro experiments. The sample size was set at *n* = 3 for rat bone defect experiments and the sample size was set at *n* = 2–3 for BM pig bone defect experiments. Error bars represent standard deviations (SD) unless otherwise stated. Significance between groups was denoted by ^*^ *p*‐value < 0.05 and ^**^ *p*‐value < 0.01. The semi‐quantitative analysis was performed on the manually acquired segmented images using ImageJ software.

## Conflict of Interest

The authors declare no conflict of interest.

## Supporting information



Supporting Information

## Data Availability

The data that support the findings of this study are available from the corresponding author upon reasonable request.
