# Supplementary material for: Oriented Cortical‐Bone‐Like Silk Protein Lamellae Effectively Repair Large Segmental Bone Defects in Pigs
Source: Adv Mater. 2025 Jan 28;37(10):2414543. doi: 10.1002/adma.202414543 (PMC11899506; doi:10.1002/adma.202414543)
Supplement: Supplementary file 1 — Supporting Information [file ADMA-37-2414543-s001.docx]

Supporting Information

Oriented Cortical-Bone-like Silk Protein Lamellae Effectively Repair Large Segmental Bone Defects in Pigs

Yajun Shuai, Tao Yang, Meidan Zheng, Li Zheng, Jie Wang, Chuanbin Mao*, Mingying Yang^*^

Yajun Shuai, Meidan Zheng, Jie Wang, Mingying Yang

Institute of Applied Bioresource Research, College of Animal Science, Zhejiang University, Hangzhou, China
E-mail: cmao@cuhk.edu.hk; yangm@zju.edu.cn

Yajun Shuai, Jie Wang, Mingying Yang
Key Laboratory of Silkworm and Bee Resource Utilization and Innovation of Zhejiang Province, Zhejiang University, Hangzhou, China

Tao Yang, Chuanbin Mao

Department of Biomedical Engineering, The Chinese University of Hong Kong, Sha Tin, Hong Kong SAR, China

Li Zheng

Guangxi Engineering Center in Biomedical Materials for Tissue and Organ Regeneration & Guangxi Collaborative Innovation Center for Biomedicine, Life Sciences Institute, Guangxi Medical University, Nanning, China

**Supplementary Experimental Section**


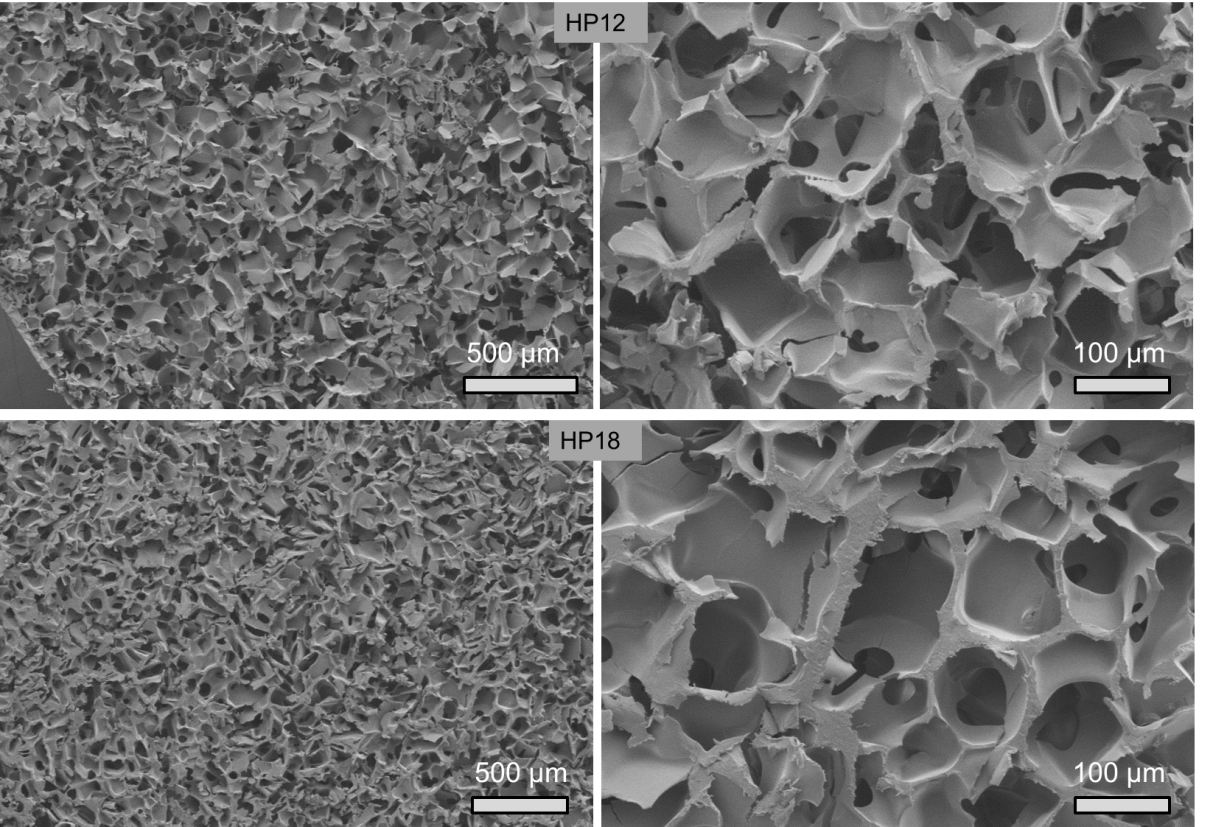


**Figure S1.** SEM of HP scaffolds at low (left) and high (right) magnification.


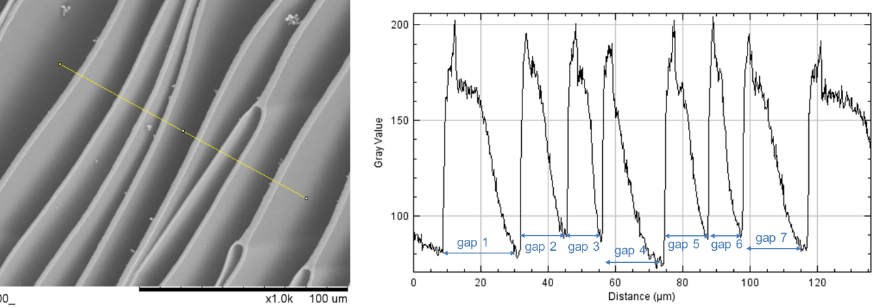


**Figure S2.** The lamella interval analysis of the LP scaffolds by ImageJ software. The right profile represents the depth change along the dashed line direction shown in the left image.


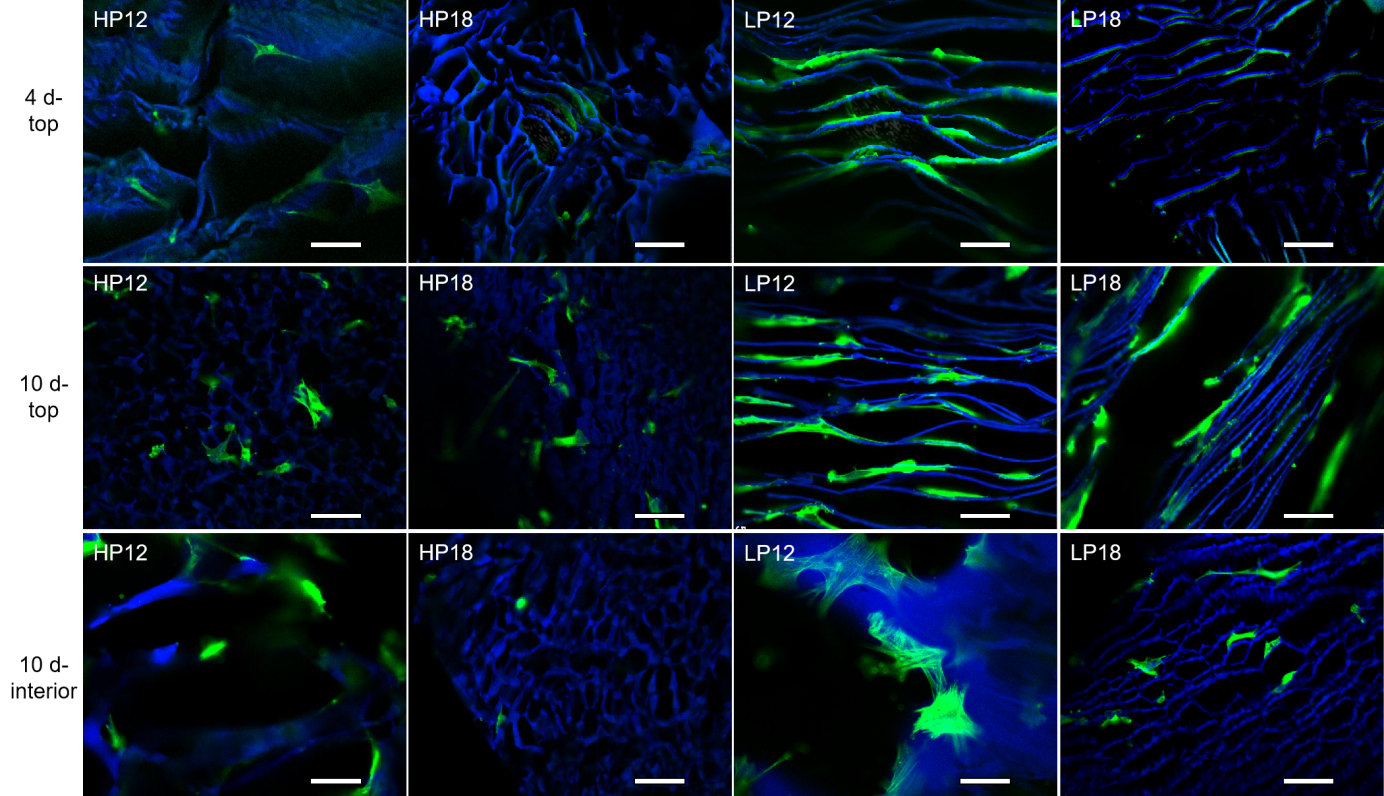


**Figure S3.** The 2D confocal imaging of rMSCs on the top or in the interior of SF-based 3D scaffolds after being cultured for 4 d and 10 d. The rMSCs on the top of lamella structures of LP scaffolds were significantly elongated with the direction of lamellar contour whereas those on the HP scaffolds were randomly oriented after 10 d of cell culture. Green fluorescence indicates FITC-labeled cells, while blue fluorescence is the autofluorescence from the SF scaffolds. HP scaffolds: honeycomb porous scaffolds. LP scaffolds: lamellar porous scaffolds. Scale bar: 100 µm.


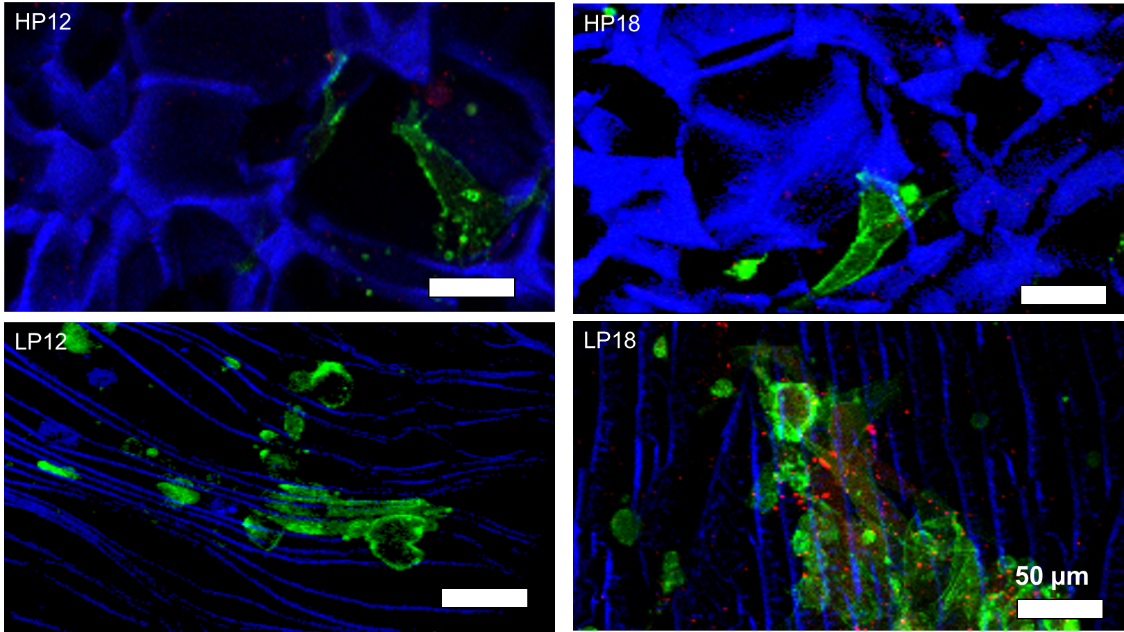


**Figure S4.** The anti-OPN immunofluorescence is stained by rhodamine-labeled anti-OPN protein (red) of hMSCs cultured in a normal medium for 14 d. Green fluorescence is FITC-labeled cells and blue fluorescence is the autofluorescence of SF scaffolds. HP scaffolds: honeycomb porous scaffolds. LP scaffolds: lamellar porous scaffolds.


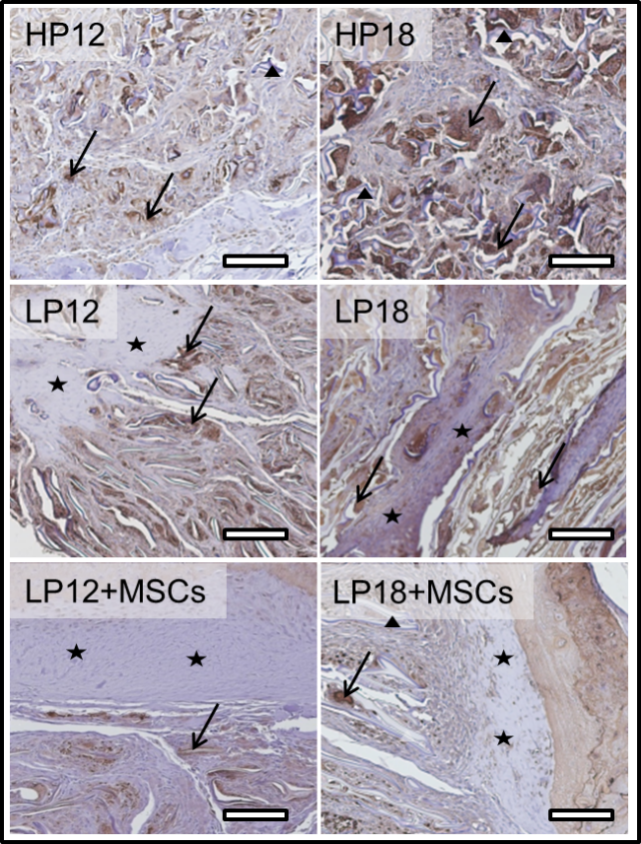


**Figure S5.** Immuno-histological characteristics for OPN of HP and LP scaffolds with or without hMSCs seeded after 3 months of surgery. Sections were counterstained with eosin after immuno-histochemistry imaging.  Arrow: OPN protein. Triangular: SF scaffolds. Star: mature bone tissue. HP scaffolds: honeycomb porous scaffolds. LP scaffolds: lamellar porous scaffolds. Scale bar: 100 µm. n=3. **P*< 0.05.


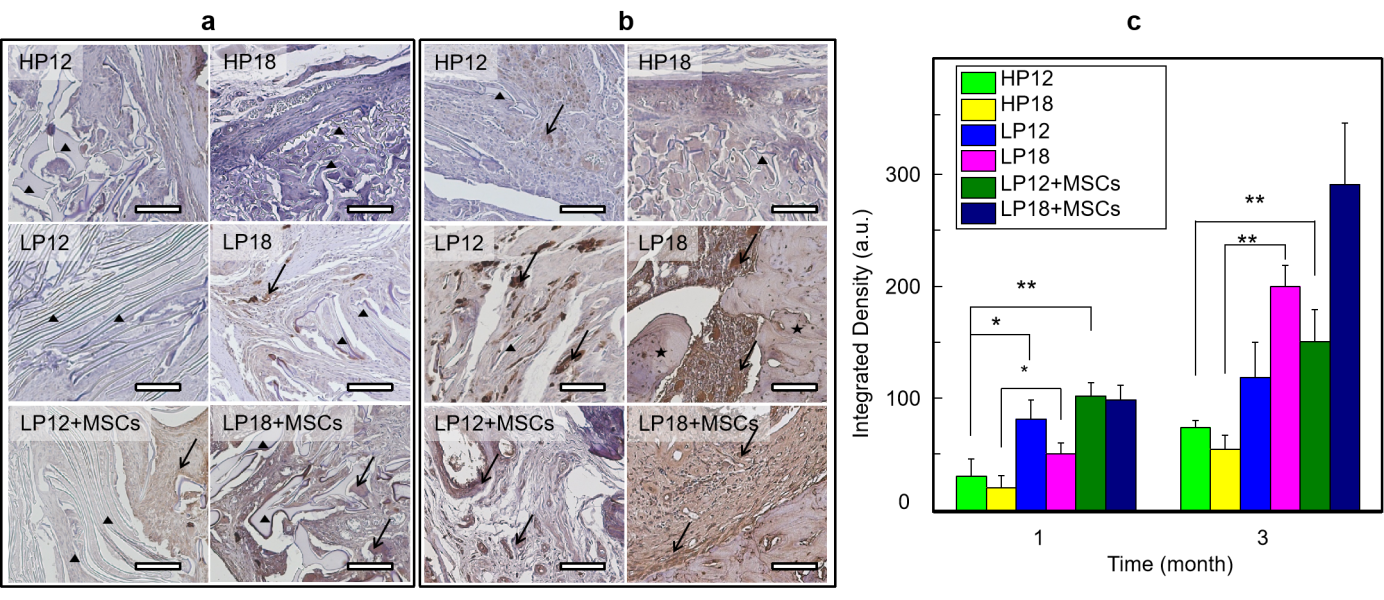


**Figure S6.** Immuno-histological characteristics for OCN of HP and LP scaffolds with or without hMSCs seeded after 1 a) and 3 months b) of surgery. Sections were counterstained with eosin after immunohistochemistry imaging. Evaluation of the effect of extracellular expression of mature osteoblasts in SF scaffolds by OCN immunohistochemical staining. The integrated densities of OCN were quantified by the ImageJ software. c) Both hMSC-seeded and non-seeded LP scaffolds displayed stronger positive immunohistochemical staining using anti-OCN antibodies than HP scaffolds after 1 month and 3 months. Arrow: OCN protein. Triangular: SF scaffolds. Star: mature bone tissue. HP scaffolds: honeycomb porous scaffolds. LP scaffolds: lamellar porous scaffolds. Scale bar: 100 µm. **P*< 0.05, ***P* < 0.01.


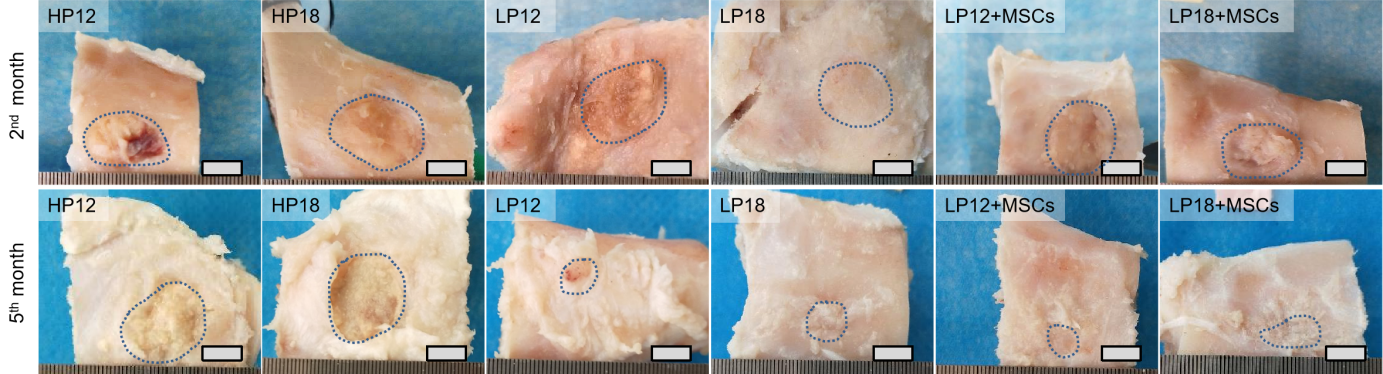


**Figure S7.** The appearance of bone tissue after 2 and 5 months of implantation. The dotted box indicates the location where the scaffold existed. Scale bar: 5 mm


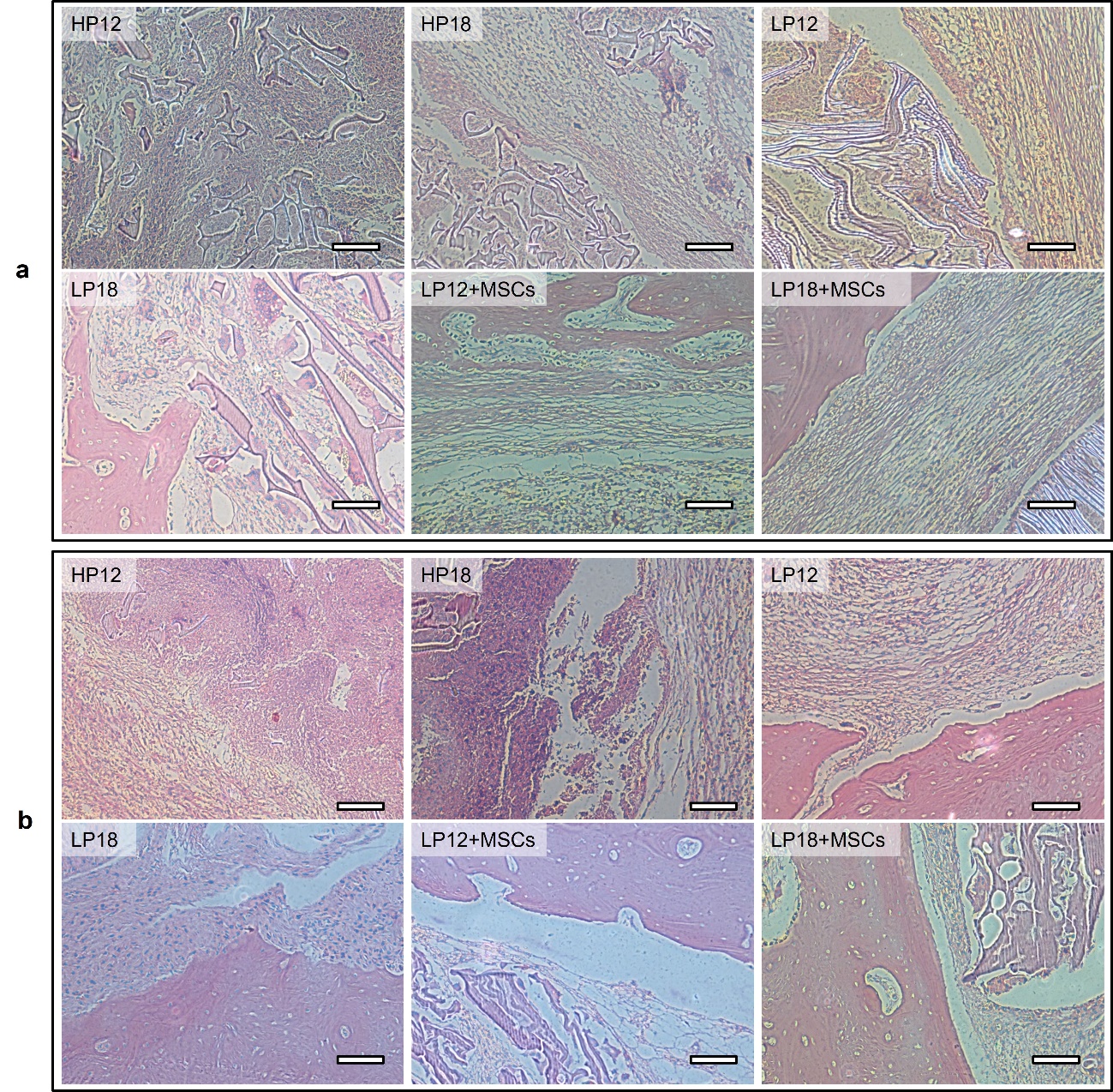


**Figure S8.** Representative H&E images of regenerated pig bone tissues after 2 months a) and 5 months b) of implantation. HP scaffolds: honeycomb porous scaffolds. LP scaffolds: lamellar porous scaffolds. Scale bar: 100 μm


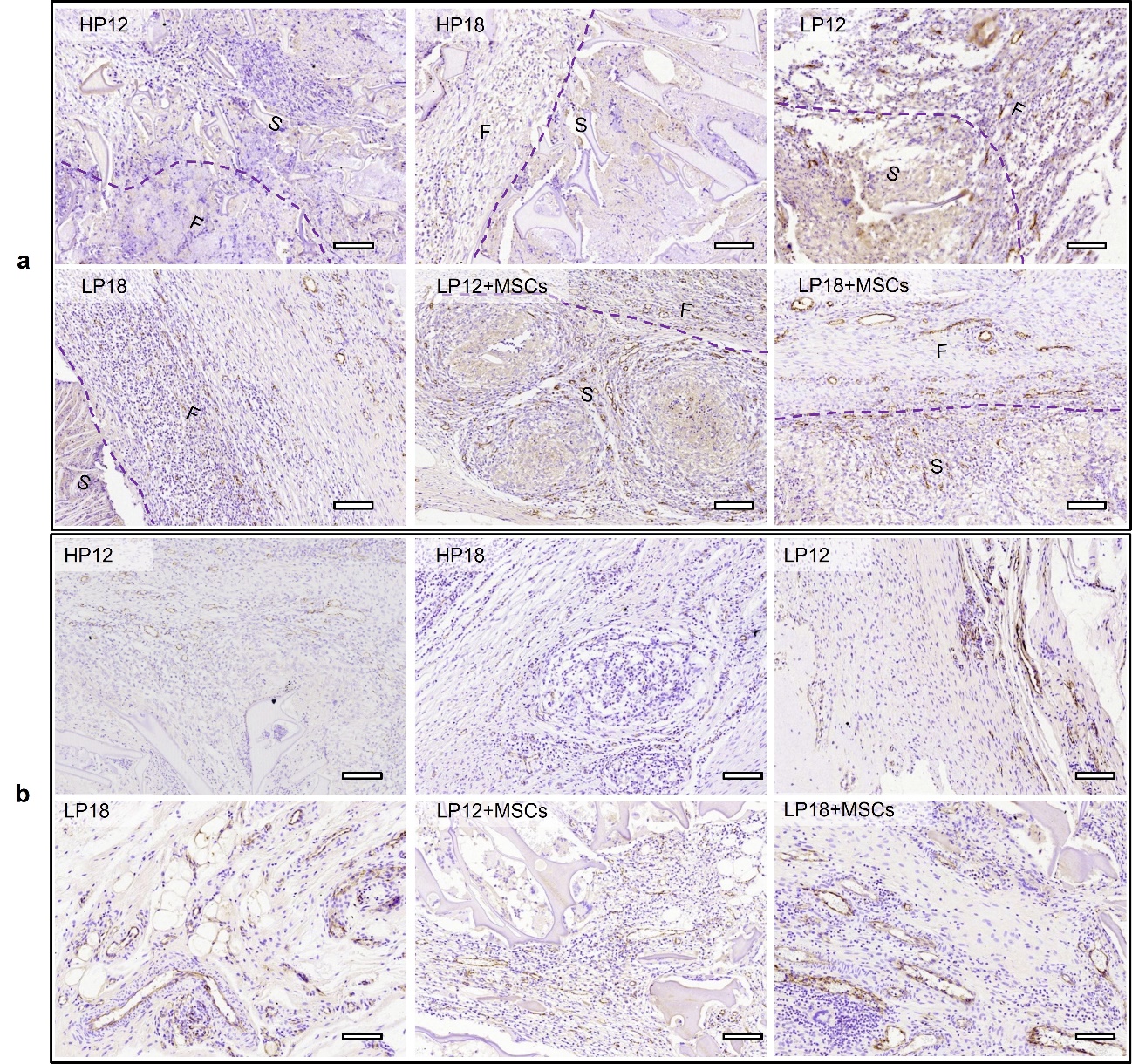


**Figure S9.** Representative CD31 staining images of regenerated BM pig bone tissues after 2 months a) and 5 months b) of implantation. The dashed lines indicate the interface between the scaffold and the new bone tissue. HP scaffolds: honeycomb porous scaffolds. LP scaffolds: lamellar porous scaffolds. S: Scaffold. F: Fibrous tissue. Scale bar: 100 μm


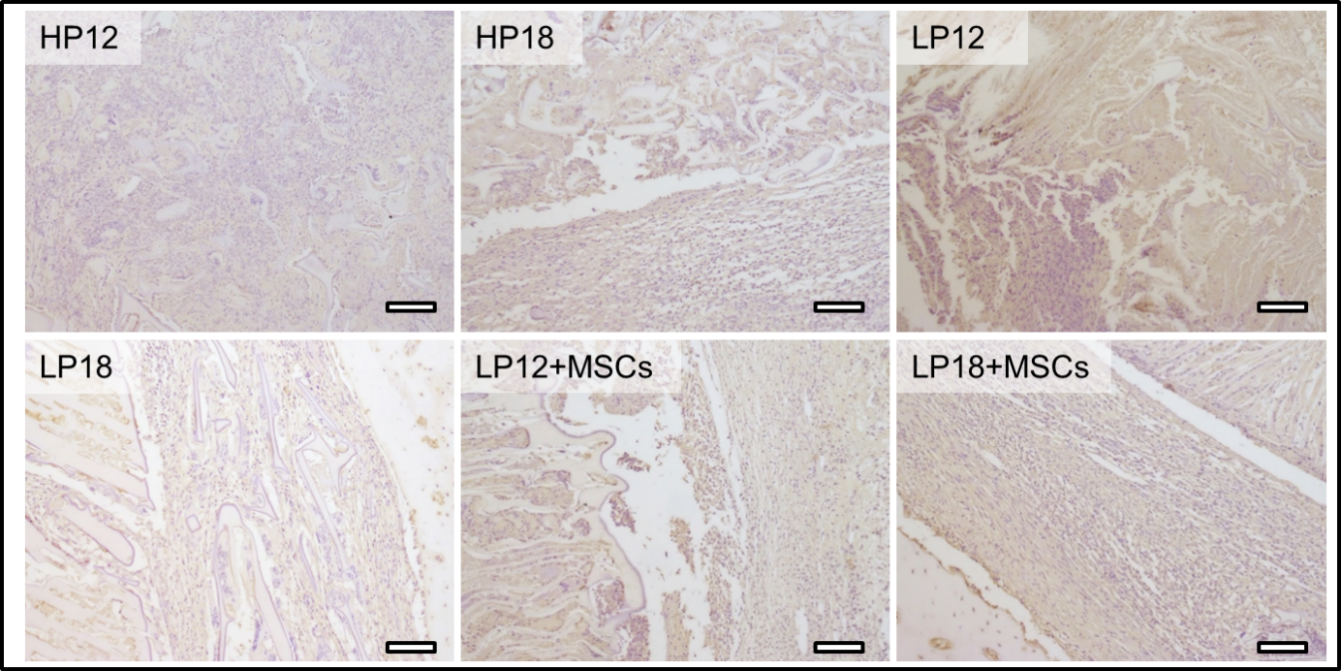


**Figure S10.** Immuno-histological characteristics for OPN in the BM pig bone defect model after 2nd month of implantation. HP scaffolds: honeycomb porous scaffolds. LP scaffolds: lamellar porous scaffolds. Scale bar: 100 μm.


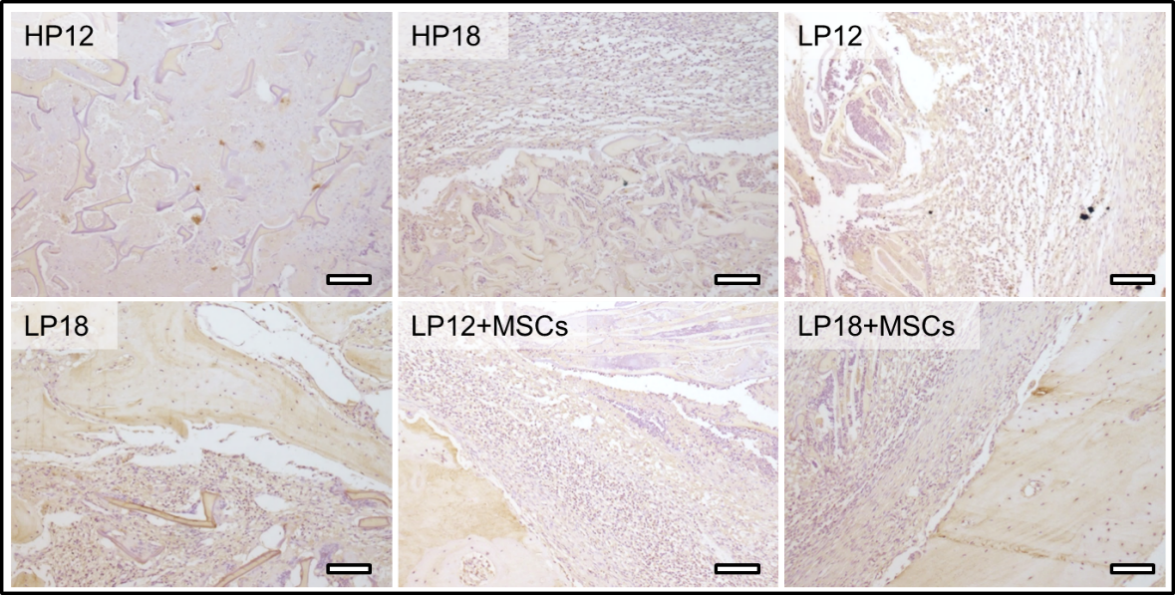


**Figure S11.** Immuno-histological characteristics for OCN in the BM pig bone defect model after 2nd month of implantation. HP scaffolds: honeycomb porous scaffolds. LP scaffolds: lamellar porous scaffolds. Scale bar: 100 μm.


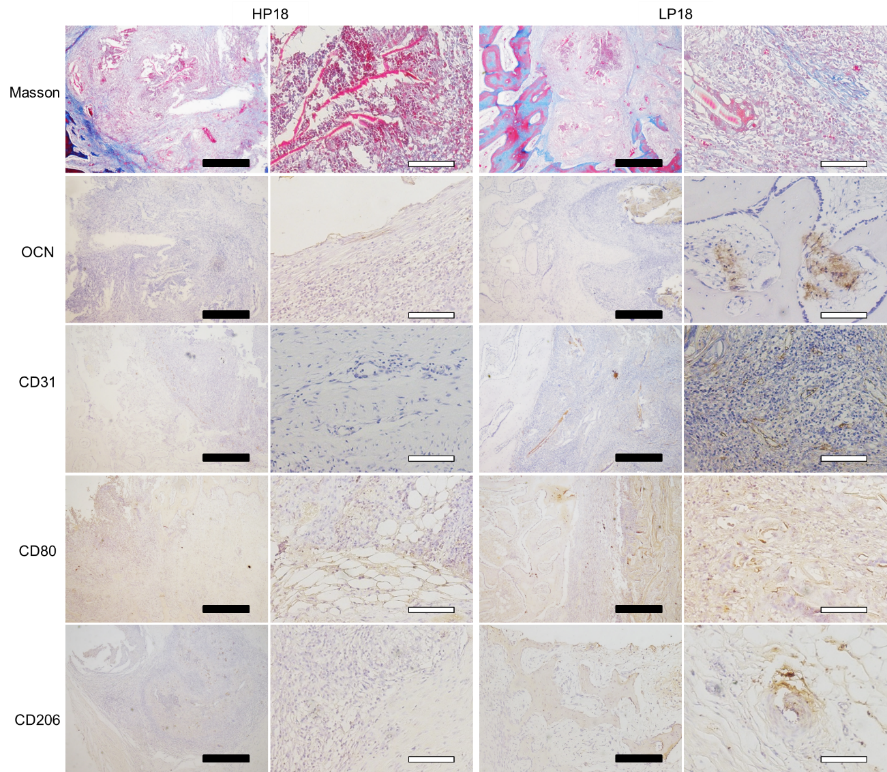


**Figure S12.** MT staining and Immuno-histological characteristics for OCN, CD31, CD80, and CD206 in pig bone defect model after 1-month implantation. Blank scale bar: 500 μm. White scale bar: 100 μm
